# Boron Vehiculating Nanosystems for Neutron Capture Therapy in Cancer Treatment

**DOI:** 10.3390/cells11244029

**Published:** 2022-12-13

**Authors:** Giorgia Ailuno, Alice Balboni, Gabriele Caviglioli, Francesco Lai, Federica Barbieri, Irene Dellacasagrande, Tullio Florio, Sara Baldassari

**Affiliations:** 1 Department of Pharmacy, University of Genova, 16147 Genova, Italy; 2 Department of Life and Environmental Sciences (DiSVA), University of Cagliari, 09124 Cagliari, Italy; 3 Department of Internal Medicine, University of Genova, 16132 Genova, Italy; 4 IRCCS Ospedale Policlinico San Martino, 16132 Genova, Italy

**Keywords:** nanoparticles, boron neutron capture therapy, thermal neutron irradiation, cancer

## Abstract

Boron neutron capture therapy is a low-invasive cancer therapy based on the neutron fission process that occurs upon thermal neutron irradiation of ^10^B-containing compounds; this process causes the release of alpha particles that selectively damage cancer cells. Although several clinical studies involving mercaptoundecahydro-*closo*-dodecaborate and the boronophenylalanine–fructose complex are currently ongoing, the success of this promising anticancer therapy is hampered by the lack of appropriate drug delivery systems to selectively carry therapeutic concentrations of boron atoms to cancer tissues, allowing prolonged boron retention therein and avoiding the damage of healthy tissues. To achieve these goals, numerous research groups have explored the possibility to formulate nanoparticulate systems for boron delivery. In this review. we report the newest developments on boron vehiculating drug delivery systems based on nanoparticles, distinguished on the basis of the type of carrier used, with a specific focus on the formulation aspects.

## 1. Introduction

Boron neutron capture therapy (BNCT) is a selective and low-invasive cancer treatment based on the neutron capture-fission response that occurs when the steady isotope boron-10 (^10^B) is irradiated with low-energy (0.025 eV) thermal neutrons or, for clinical studies, higher energy epithermal neutrons (10,000 eV), which may penetrate into thick tissues and the skull, a critical feature for BNCT success [1,2].

As a consequence of neutron irradiation, unstable ^11^B isotopes undergo a neutron fission process leading to the release of a high-linear energy transfer of alpha particles (^4^He), lithium-7 ions (^7^Li), and gamma radiations. The alpha particles selectively damage cancer cells, provided that sufficient ^10^B has accumulated within the tumor area; indeed, alpha particles are characterized by very short path lengths (5–9 µm) and, therefore, their destructive effects are limited to boron-containing cells [1,3]. To be effective, about 20 µg of ^10^B per gram of tumor tissue, equivalent to about 10^9^ atoms/cells, have to be selectively delivered to the tumor [1,2], and a sufficient dose of neutrons must penetrate and be absorbed by the cells to initiate the fission reaction [4,5].

Clinical interest in BNCT is primarily directed to high-grade gliomas, head and neck tumors [6,7,8,9], and, to a lesser extent, cutaneous and extra-cutaneous melanomas [10,11,12].

After the first BNCT clinical trial, conducted by Farr et al. in 1951 at the Brookhaven Graphite Research Reactor, involving patients with glioblastoma [13], the number of trials for BNCT increased, especially in the first years of the XXI century [2]. The general requirements for a BNCT delivery agent are: high tumor uptake, low intrinsic toxicity, and low normal tissue uptake, ideally featuring tumor:normal tissue and tumor:blood boron concentration ratios higher than 3:1; moreover, the ideal BNCT agents should exhibit persistence in the tumor for at least several hours and relatively rapid clearance from blood and normal tissues [1].

The boron agents currently used in the clinics are sodium mercaptoundecahydro-*closo*-dodecaborate (Na_2_B_12_H_11_SH), known as sodium borocaptate (BSH), and (L)-4-dihydroxy-borylphenylalanine, commonly known as boronophenylalanine (BPA).

BSH, a polyhedral borane anion synthesized for the first time by Miller et al. in 1963 [14], was initially used in clinics to treat patients with high-grade gliomas [15,16,17]. Clinical trials using BSH have involved patients affected by glioblastoma [18] and other high-grade gliomas [19], meningiomas [20], head and neck tumors [21,22], liver metastases of colorectal adenocarcinoma [23], and hepatocellular carcinoma [24].

BPA is a boron-containing amino acid that was initially employed to treat patients with cutaneous melanomas, based on the assumption that this molecule would be preferentially uptaken by melanin-synthesizing cells [25]. Soon thereafter, it was demonstrated that the compound accumulated also in other histological types of tumors, including a rat brain tumor [26]; hence, since the beginning of the century, BPA and its fructose-complex (BPA-fr, significantly improving BPA water solubility) [27] have been tested in a number of clinical trials for the treatment of glioblastoma [21,28,29,30,31,32,33,34], meningiomas [20], head and neck tumors [22,35,36,37,38], melanoma [39,40], hepatocellular carcinoma [24], and extramammary Paget’s disease [11]. Importantly, in vitro and in vivo BNCT studies indicate that glioma stem-like cells are able to uptake BPA or other boron agents with high efficacy, in some cases even higher than that observed in the differentiated cell counterpart [41]. Nowadays, tumor stem cells are considered the main pharmacological target to eradicate the most malignant tumors, due to their chemo- and radiotherapy resistance [42]; this evidence further supports the high clinical potentiality of BNCT.

Despite the promising results obtained from clinical trials encouraging further investigation of BNCT as an effective modality for cancer therapy, the limited tumor/normal tissue ratios of boron in patients treated with BPA and BSH calls for the development of more effective and selective boron delivery agents. BNCT effectiveness largely relies on the accumulation of a high amount of ^10^B in tumor cells; therefore, the development of novel boron delivery agents, able to vehiculate high boron amounts selectively to the tumor cells, is one of the most important needs for BNCT success. Boron delivery agents can be classified into three categories: small molecules enriched in ^10^B, boron compound conjugates, and boron-containing nanoparticles (NPs) [43,44,45].

This article reviews examples of nanosystems for BNCT application developed so far, classified by the type of carrier used, detailing the formulation aspects crucial for the success of these boron delivery systems, highlighting the most promising ones, on the basis of the drug loading and the development advancement. Table 1 summarizes the liposomal formulations for BNCT applications reviewed in this article, while Table 2 displays the other nanosystems, classified by the type of NP, that have been proposed as BNCT tools. Among the high number of literature works in the field, we decided to focus on the most recently published papers.

## 2. Nanoparticles for BNCT

### 2.1. Liposomes

Liposomes, round-shaped vesicles composed of an aqueous core surrounded by a phospholipid double-layer, are generally endowed with high biocompatibility/biodegradability and low toxicity.

Several studies for the encapsulation of boron compounds into liposomes have been conducted since the last decade of the past century [46,47,48]. In more recent years, Kueffer and coworkers [49] developed a liposomal formulation vehiculating boron in both the aqueous interior and the lipid bilayer: these liposomes, prepared by probe sonication and composed of 1,2-distearoyl-*sn*-glycero-3-phosphocholine (DSPC) and cholesterol, encapsulated the ammonium derivative Na_3_[1-(2′-B_10_H_9_)-2-NH_3_B_10_H_8_], which was dissolved in the hydrating solution, and embedded in the liposome membrane the lipophilic K[*nido*-7-CH_3_(CH_2_)_15_-7,8-C_2_B_9_H_11_], which was added to the lipid mixture. The obtained liposomes exhibited an average diameter ranging from 109 to 134 nm and negative zeta potential (−76.4 ± 1.1 mV), measured by dynamic light scattering (DLS). Biodistribution studies were performed on female mice inoculated with breast cancer cells, following a “double-injection” protocol: by administering two doses of liposomes 24 h apart, the authors observed that the tumor:blood ratio continued to increase up to 96 h after the first injection, providing a wide therapeutic window available for neutron irradiation. As a result, tumor growth was slowed down after double irradiation of mice. However, the authors also pointed out some limitations in their study, such as the limited dose of radiation mice were exposed to, and the high mass of the tumors developed in their model, considering that larger tumors retain boron compounds on the peripheries, diminishing the apparent boron concentration in the tumor and preventing its eradication, as confirmed in a following study [50].

Two novel synthesized boron compounds, *o*-closocarboranyl β-lactoside (LCOB) and 1-methyl-*o*-carboranyl-2-hexylthioporphyrazine (H2Pz-COB) were introduced in the lipid layer of liposomes by Altieri and his group [51]. Three liposomal formulations differing for the surface charge were prepared: cationic liposomes obtained from a mixture of 1,2-dioleoyl-3-trimethylammonium-propane (DOTAP)/1,2-dioleoyl-*sn*-glycero-3-phosphocholine (DOPC)/1,2-dioleoyl-*sn*-glycero-3-phosphoethanolamine (DOPE), anionic liposomes composed of 1,2-dioleoyl-*sn*-glycero-3-phosphate (DOPA)/DOPC/DOPE, and the zwitterionic formulation including DOPC/DOPE. The three formulations were loaded with LCOB or H2PzCOB using the coextrusion and sonication procedure, respectively. LCOB-loaded liposomes exhibited an average diameter of 150–160 nm, while H2PzCOB-loaded liposomes showed higher dimensions (ranging from 240 to 450 nm) and polydispersity index. Contrary to the LCOB-loaded liposomes, H2PzCOB-loaded liposomes tended to grow and aggregate, leaking boron within a few days from preparation. By measuring the α particle emission, the ^10^B uptake was studied in vitro on rat colon carcinoma and murine melanoma cells, evidencing that in both cell lines the cationic liposomes featured the highest boron uptake regardless of the encapsulated boron compound. Interestingly, despite the stability issues, H2PzCOB-loaded cationic liposomes exhibited the highest boron release in colon carcinoma cells.

An innovative approach to target glioblastoma was described by Chen and his group [52], developing a liposomal formulation with a double anti-cancer effect and functionalized with the internalizing-RGD (iRGD) peptide. The authors developed liposomes encapsulating both a complex of doxorubicin with 1-bromomethyl-*o*-carborane (DOX-CB), which should translocate into the nucleus thanks to the DOX nuclear tropism, and the CD47 gene-targeted CRISPR-Cas9 gene knock-out plasmid (pDNA) that, by directly knocking out the CD47 gene, promotes the macrophage-mediated phagocytosis of cancer cells. Cationic liposomes (DOX-CB@lipo) were prepared by hydration of a thin film formed by DOTAP, DOPE, cholesterol, 1,2-distearoyl-*sn*-glycero-3-phosphoethanolamine-N-[amino(polyethyleneglycol)-2000] (DSPE-PEG) and DOX-CB, followed by probe sonication. DOX-CB@lipo-pDNA were obtained by incubation of DOX-CB@lipo with pDNA, based on the principle of charge interactions, and finally, the functionalization with iRGD was carried out by incubation. The obtained liposomes exhibited an average diameter of 150 nm and a positive zeta potential of about 20 mV. By fluorescence microscopy, western blot analysis, and flow cytometry, the authors demonstrated the superiority of this formulation, compared to the commercial lipofectamine 2000, in inhibiting the CD47 expression in glioma cells. The nuclear uptake of boron in cells incubated with DOX-CB@lipo-pDNA-iRGD, measured by inductively coupled plasma mass spectrometry (ICP-MS), was 19.56 ± 8.05 ng/10^6^ cells, while the boron uptake by the whole cells was 41.28 ± 13.87 ng/10^6^ cells, which are ideal values for BNCT applications. The efficacy of this novel BNCT agent was assessed by observing the prolonged survival of glioma-bearing mice undergoing thermal neutron irradiation after receiving DOX-CB@lipo-pDNA.

To improve BPA and BPA-fr pharmacokinetics, several research groups investigated the possibility of using liposomal carriers. Interestingly, by exploiting high-resolution ^1^H- and ^13^C-NMR, Martini et al. [53] studied the molecular interactions occurring during the insertion of the hydrophobic BPA within the membrane of cationic liposomes composed of DOTAP and DOPE, showing that the tumor-targeting portion of BPA was not masked by liposome insertion, thus this approach might provide a good boron carrier in BNCT.

To increase ^10^B uptake in liver metastatic tissue, Pavanetto et al. [54] prepared both conventional liposomes, composed of phosphatidylcholine and cholesterol, and stabilized liposomes including monosialoganglioside (GM1) or DSPE-PEG, exploiting the reverse-phase method followed by extrusion, as already described in their previous work [120]; each formulation was loaded with BPA-fr, locating in the aqueous core, or with both BPA-fr and neat BPA, embedding in the lipid bilayer thanks to its lipophilicity. The obtained PEGylated liposome diameter (about 156 nm) was smaller than the one of conventional (171 nm) and GM1-covered (195 nm) liposomes. By evaluating the encapsulation efficiency (EE), the authors highlighted that the ^10^B content per lipid unit (determined by ICP-MS) increased when GM1 or DSPE-PEG were added to the lipidic bilayer, and that liposomes carrying both BPA-fr and BPA exhibited a drug content 4-fold higher than liposomes encapsulating BPA-fr only. ICP-MS and autoradiographic ex vivo analysis performed on rat liver metastases evidenced that PEGylated liposomes led to blood and tissue boron concentrations suitable for BNCT application, contrary to conventional and GM1-containing liposomes.

With the aim of improving the delivery and retention of boronated compounds in tumor tissue, Luderer et al. [55] developed temperature-sensitive liposomes (TSL) loaded with BPA-fr and B-381, a boronated 2-nitroimidazole derivative previously synthesized by the same group [121]. The TSL, composed of DPPC/DSPC/DSPE-PEG/cholesterol, were prepared by thin film hydration using an aqueous solution of BPA-fr (30 mg/mL, pH 7) or a solution of B-381 in sodium citrate buffer (40 mg/mL, pH 4). Interestingly, the presence of cholesterol resulted to be fundamental to obtain the temperature-controlled release of B-381. The liposomes were characterized by size (133.6 nm for B-381 TSL and 140.0 nm for BPA-fr TSL) and zeta potential (−5.59 mV and −4.36 mV, respectively); the EE was about 4.5–5% for both formulations, with a boron content ranging from 1317 to 1505 ppm, measured by inductively coupled plasma optical emission spectrometry (ICP-OES). The TSL formulations showed low BPA-fr and B-381 release at 37 °C (19 and 20%) and high release at 42 °C (77% and 90%, respectively). The specific boron delivery was investigated on mice bearing bilateral gliomas and, by ICP-OES, BPA-fr TSL and B-381 TSL showed 8.4-fold boron accumulation in the hyperthermia-treated tumor area, compared to the tumor tissue left at physiologic temperature (692 ± 99 ppb and 82 ± 20 ppb, respectively); notably, in the case of B-381 TSL, the authors observed longer boron retention in the tumor, which might be explained by considering that in a hypoxic microenvironment B-381, as other 2-nitroimidazole derivatives, forms intracellular protein conjugates leading to its preferential accumulation and retention.

Numerous research groups focused their activity on the development of liposomal formulations to improve the delivery of the already-mentioned BSH compound. For example, Mehta et al. [56] studied the biodistribution of conventional and PEGylated liposomes encapsulating BSH, while Yanagië et al. [57,58] encapsulated a cesium salt of BSH (Cs_2_^10^B_12_H_11_SH) into multilamellar immunoliposomes ([^10^B]immunoliposomes) for pancreatic cancer targeting, justifying the choice of preparing multilamellar vesicles by considering that they present a larger lipophilic compartment than unilamellar ones, and are therefore more suitable for the inclusion of lipophilic active compounds [122]. 

To target BSH to tumors, Maruyama and his group developed PEGylated liposomes functionalized with transferrin (TF-PEG liposomes) [59], exploiting the overexpression of the TF receptor in tumor cells [123]. The liposomes, characterized in a previous work [124], were prepared from DSPC, cholesterol, DSPE-PEG, and 1,2-distearoyl-*sn*-glycero-3-phosphoethanolamine-N-[carboxy(polyethylene glycol)-2000] (DSPE-PEG-COOH) by reverse phase evaporation using a 125 mM BSH aqueous solution; for TF coupling, the authors exploited the formation of amidic bonds with the DSPE-PEG-COOH carboxylic group, activated with 1-ethyl-3-(3-dimethylaminopropyl)-carbodiimide (EDC) and *N*-hydroxysulfosuccinimide (S-NHS). The obtained TF-PEG liposomes exhibited an average diameter of 122.8 ± 31 nm and an EE of 6–8%; the ^10^B content per µmol lipid was 26–30 µg, as calculated from the results of lipid concentration measurement by phosphorus assay and ^10^B concentration measurement by a microwave-induced plasma mass spectrometer (MIP-MS). The specificity of the TF-receptor mediated binding was verified in vitro in colon carcinoma cells, while an ex vivo biodistribution study, conducted on colon carcinoma-bearing mice, revealed, by MIP-MS, prolonged residence of TF-PEG liposomes in the circulation. TF-PEG liposomes led to 35.2 µg/g of ^10^B accumulated in the tumor 24 h after injection, which was 2.1-fold higher than that of bare liposomes. Moreover, the presence of TF on the liposome surface increased the concentration and retention time in the tumor, with the AUC_tumor_ of TF-PEG liposomes 1.4 times higher than that of untargeted PEGylated liposomes. Finally, the authors showed that thermal neutron irradiation of colon carcinoma-bearing mice previously injected with TF-PEG liposomes exhibited decreased tumor growth rate, up to complete disappearance 12 days after treatment, resulting in 100% animal survival.

Epidermal growth factor receptor (EGFR) is a transmembrane tyrosine kinase overexpressed in human glioblastoma, thus representing a promising target for BSH selective delivery. Feng et al. [60] prepared, by the reverse phase method, EGFR-targeting immunoliposomes composed of DOPC, 1,2-dioleoyl-*sn*-glycero-3-phospho-(1′-rac-glycerol) (DOPG), 1,2-dioleoyl-*sn*-glycero-3-[(*N*-(5-amino-1-carboxypentyl)iminodiacetic acid)-succinyl] nickel (DOGS-NTA-Ni), cholesterol and DSPE-PEG, using a 50 or 300 mM BSH solution. To conjugate rat or mouse anti-EGFR monoclonal antibodies (mAb) to the nickel-liposomes, the recombinant ZZ-His (IgG Fc-binding motif), able to bind to nickel, was used as an adaptor. The obtained liposomes exhibited a 130 nm average diameter and a negative zeta potential (about −25 mV). Using an anti-BSH mAb, the authors examined the in vitro delivery of ^10^B from the immunoliposomes to different tumor cell lines: BSH was detected in U87 glioblastoma cells overexpressing WT EGFR or the tumorigenic mutation EGFRvIII, but not in primary astrocytes and the parental U87 cells, lacking EGFR expression. The dose-dependent effect of the anti-EGFR antibody on the efficiency of ^10^B delivery was confirmed by ICP-OES. Biodistribution studies were conducted on U87-injected mice receiving liposomes fluorescently labeled with 25-[*N*-[(7-nitrobenz-2-oxa-1,3-diazol-4-yl)-methyl]amino]-27-norcholesterol (NBD-cholesterol): in brain slices from mice treated with the labeled immunoliposomes, fluorescence was detected in the tumor and, weakly, in related blood vessel walls, indicating crossing ability from blood to tumor tissue, while no fluorescence was observed in normal tissues. The presence of BSH in tumor tissues was further confirmed by immunohistochemical analysis.

A different strategy to vehiculate BSH to cancer cells was realized by Ueno and his group [61], who were the first to synthesize *nido*-carborane lipids from different phospholipids used to prepare liposomes that, when administered to tumor-bearing mice before irradiation, inhibited the tumor growth and mortality.

Similarly, Koganei and his group [62] exploited a boronated lipid obtained from the conjugation of BSH to DSPC (called DSBL lipid) [125] to prepare boronated liposomes and, to increase the boron content, BSH was also loaded in the liposome aqueous core. The particles, composed of various ratios of DSBL, DSPC, cholesterol, and DSPE-PEG, were prepared by the reverse phase evaporation method using 125 mM BSH aqueous solution and exhibited a 127 ± 0.71 nm average diameter and high boron concentration (5000 ppm) with a boron/phosphorous (B/P) ratio of 2.6, determined through ICP-OES. Biodistribution studies on healthy mice evidenced that the formulation containing a 10% mol ratio of DSBL was the most suitable for boron delivery, due to its higher blood boron concentrations and longer circulation in the blood. The authors also performed an in vivo tracking of the 10% DSBL liposomes loaded with a magnetic resonance imaging (MRI) contrast agent, Magnetoscope, injected in colon carcinoma-bearing mice: this study evidenced that the signal intensity of the tumor area increased 24 h after injection, reaching the highest signal intensity detected after 36 h, with boron concentrations reaching 174 ppm at the maximum injected dose (50 mg B/kg). Noteworthy, even at the low boron dose (15 mg B/kg), the concentration in the tumor tissue was sufficiently high to induce an efficient BNCT effect on cancer cells, as confirmed by the decreased tumor growth in mice injected with BSH encapsulating 10% DSBL liposomes and undergoing thermal neutron irradiation. Therefore, due to the high B/P ratio and the excellent boron delivery efficacy to tumors, this novel liposomal formulation effective dose should cause very little liver toxicity.

In a following work, the same authors investigated the effects of counter cations of boron clusters on liposome formation, to overcome osmotic pressure limitations occurring when encapsulating boronated compounds [63], finding out a possible beneficial effect of the spermidinium counter cation on the particle encapsulation of *closo*-dodecaborate. 

Recently, an interesting study aimed at improving BSH EE has been published by Shirakawa and his group [64]. Liposomes composed of DSPC and cholesterol at a constant ratio were prepared by the thin film hydration method, and BSH was loaded by following different procedures: ultrasonic treatment, freeze–thaw cycles, or reverse phase evaporation method. Since the loading method did not significantly affect the boron EE, determined by ICP-OES, the effects of the concentrations of lipid and BSH aqueous solutions were investigated using the freeze-thaw cycles. After optimizing both lipid and BSH concentrations (150 mg/mL and 28 mg/mL, respectively) and obtaining a formulation with high boron content, the authors also prepared PEGylated liposomes and demonstrated their high in vitro stability, with no significant BSH leakage (less than 10% in 72 h).

### 2.2. Elemental Boron Nanoparticles

Elemental boron particles represent a promising new drug delivery system to target boron for the treatment of cancer through BNCT, owing to the high content of boron atoms in one particle [65]. However, elemental boron NPs lack active tumor-targeting properties and, in aqueous solutions, tend to aggregate over time. To overcome these issues, Zaboronok et al. [66] focused on the search for a suitable polymeric stabilizer allowing high NP loading capacity and featuring reactive functional groups for biomolecular vector attachment, identifying hydroxyethyl cellulose (HEC) as a promising candidate. The boron NPs, prepared following a patented procedure [126], were stabilized with 0.3% HEC (1000 kDa) aqueous solution by continuous stirring. Size (about 25–28 nm) and zeta potential (+45.6 mV) of the HEC-boron NPs, monitored through DLS, resulted stable for over 90 days. Then, in vitro tests on different glioma cell lines evidenced, after neutron irradiation, reduced cell proliferation. Nevertheless, a limitation of this study, underlined by the authors, is the lack of boron concentration determination and the consequent absence of calculations regarding the boron-dependent absorbed dose during irradiation: indeed, the authors declared that the ICP-OES measurement was not possible because of the incomplete dissolution of the stabilized NPs in the acidic medium necessary for sample preparation, and the microwave digestion was not possible because of logistical restrictions. 

Noteworthy, in a very recent work, Pastukhov and coworkers [67] proposed a simple procedure for producing elemental boron NPs by laser ablation in aqueous medium followed by PEG grafting, to improve their stability. As stated by the authors, the described procedure might provide the maximal concentration of boron atoms for BNCT and avoid any toxic contamination, representing an innovative boron NPs fabrication procedure for biomedical applications.

### 2.3. Iron Oxide Nanoparticles

The design of bimetallic NPs, containing both Fe and B, provides a desirable set of functions for advanced BNCT treatments, allowing MRI localization of B-containing compounds, their magnetophoretic accumulation to increase B uptake in the tumor tissue, and increased tissue permeation resulting from high temperatures induced by magnetic hyperthermia [74,127].

Increasing interest is directed to the design and construction of hybrid nanocomposites, involving the anchoring/formation of superparamagnetic iron oxide NPs (SPIONs) on heterogeneous nanomaterials, that might generate magneto-manipulable nanovehicles preventing the aggregation of iron oxide NPs and implementing multifunctional properties within a single system [128]. In this context, Wang and his group [68] developed hybrid nanocomposites consisting of boron nitride nanospheres (BNNS) coated by Fe_3_O_4_ NPs. The BNNS were prepared via a carbon-free chemical vapor deposition method, exploiting trimethoxyborane and ammonia as reactants, then the Fe_3_O_4_@BNNS nanocomposites were obtained by an ethanol-thermal method. After investigation of the morphology and structure of the nanocomposites through transmission electron microscopy (TEM), X-ray diffraction (XRD), scanning electron microscopy (SEM), and Fourier transform infrared (FTIR) spectroscopy, the authors confirmed the biocompatibility of this system on different cell lines through an MTT assay.

An opposite possible strategy, proposed by other research groups, is to graft carborane compounds to the surface of iron oxide NPs exploiting the reaction with amino groups introduced on the NPs surface using (3-aminopropyl)trimethoxysilane (APTMS) [69,70].

Nanocomposites potentially applicable for BNCT were developed also by Korolkov and his group [71]. In this study, Fe_3_O_4_ NPs, prepared by an alkaline precipitation method, were coated with tetraethoxysilane and (3-glycidylpropyl)trimethoxysilane, with the aim of stabilizing the system against precipitation and introducing epoxy active groups for the following conjugation with isopropyl-*o*-carborane: the obtained NPs had an average diameter of about 60 nm and exerted low cytotoxicity on different cancer cell lines. In a very similar work published by the same authors quite at the same time, the iron oxide NPs were functionalized with the same carborane compound by previously coating the NP surface via subsequent reactions involving tetraethoxysilane and 3-(trimethoxysilyl)propyl methacrylate, creating C=C double bonds for further graft polymerization of glycidyl methacrylate [72].

Among the carborane derivatives used in combination with magnetic NPs, carboranylphosphinates stand out due to their high boron content and good affinity for magnetic NPs [129]. In order to develop biocompatible agents for cell labeling used for both tracking purposes and BNCT, Oleshkevich et al. [73] prepared boron cluster-magnetic nanohybrids (1-MNPs) coated with *m*-carboranylphosphinate by the classic co-precipitation synthesis [130]. NPs uptake in human brain endothelial and glioblastoma cells was assessed by magnetism measurements and TEM, while in vitro BNCT studies showed a consistent reduction of proliferation rate when cells were irradiated after incubation with 1-MNPs. A preliminary in vivo toxicity test evidenced no major signs of toxicity within 10 days.

An innovative strategy to produce bimetallic NPs coated by a shell of polyvinyl pyrrolidone (PVP) was recently described by Torresan et al. [74], using a one-pot approach based on laser ablation in liquid [75]. The authors assessed that this method was easy, cost-effective, scalable, and avoided the introduction of chemical contaminants, only involving the bulk metal components and a solution of PVP in acetone. To prepare the Fe-B NPs, laser ablation was conducted with 1064 nm (6 ns) laser pulses under argon flux, to prevent Fe and B oxidation, and the polymeric coating was applied by dispersing the NPs in the PVP solution. Through ICP-MS, the final NPs composition resulted in a Fe/B atomic ratio of 1.0:0.4; the hydrodynamic size was 64 ± 40 nm, and the PVP-coating was further confirmed by FTIR spectroscopy. Fe–B NPs resulted to be biocompatible with three different mouse cell lines and human peripheral blood mononuclear cells, with a dose-dependent internalization in all tested cells; as expected from phagocytes, monocytes, and macrophages from blood mononuclear cell culture exhibited the highest uptake. In vivo biodistribution studies performed on healthy mice evidenced, through MRI, that Fe–B NPs had longer circulation time than the benchmark Endorem, used as a comparison, and did not immediately accumulate in the spleen and kidneys, avoiding the risk of relevant side effects.

In recent years, new nanocomposites combining Fe, B, and Gd have been developed for more efficient neutron capture therapy, with the benefit of using both boron and gadolinium compounds due to the added diagnostic properties and synergetic effect in the neutron capture relations [131,132] and possible simultaneous MRI during neutron capture therapy. In this context, Icten and his group [76] developed Fe-Gd-B triple nanoplatforms using simple and biocompatible inorganic chemicals such as boric acid (BA) or borax (BX), Gd(III) salts, and iron oxide. In particular, Fe_3_O_4_-GdBO_3_ nanocomposites of about 100–150 nm diameter were prepared by using bare or PEGylated Fe_3_O_4_ NPs as the core, while vaterite-type orthoborate [Gd_3_(B_3_O_9_)] or triclinic-GdBO_3_, respectively obtained when using BA or BX, constituted the external borate layer. Following the success of this preliminary research, the same authors investigated the possibility of bioconjugating the nanocomposites in two different ways [77]: functionalization with citric acid (CA), to improve water solubility and allow conjugation with different types of molecules; and coating with fluorescein isothiocyanate-doped silica (FITC-SiO_2_) for conjugation with folic acid, enabling optical imaging and cancer targeting. For CA conjugation, the nanocomposites were dispersed in a mixture of *o*-dichlorobenzene and dimethylformamide, then CA was added; in the second case, after FITC-SiO_2_ coating following a procedure previously described [133], amino groups were introduced using (3-aminopropyl)triethoxysilane (APTES) and folic acid was conjugated through an amidic bond in the presence of EDC and NHS. Both formulations displayed negative zeta potential values between −15 and −26 mV; after confirming the CA and folic acid coating through FTIR spectroscopy and UV–visible spectroscopy, respectively, the loading efficiencies of B and Gd were determined as 2.5 wt% (2.7 × 10^14^ atom ^10^B/μg composite) and 30 wt% (1.8 × 10^14^ atom ^157^Gd/μg composite) in the CA-coated nanocomposites, 1.6 wt% B (1.8 × 10^14^ atom ^10^B/μg composite) and 20 wt% Gd (1.2 × 10^14^ atom ^157^Gd/μg composite) in the folic-acid nanocomposites. The loading efficiency results make both formulations suitable for BNCT and GdNCT applications, and also for a combined GdBNCT, being Gd/B > 3. Finally, the selective uptake of folic acid-coated nanocomposites was assessed in human pancreatic, lung, and cervical cancer cells by fluorescence imaging and fluorescence-activated cell sorting (FACS) analysis, while the CA-conjugated nanocomposites exerted the suppression of the pancreatic cancer cell growth in a dose-dependent manner.

Moreover, Korolkov and his group [78] developed a Fe-B-Gd nanosystem obtained by B and Gd immobilization on the Fe_3_O_4_ NP surface, through the alkaline coprecipitation method. The coating was performed by reacting the iron oxide NPs with 3-(trimethoxysilyl)propyl methacrylate followed by acrylic acid, thus introducing the carboxylic groups that, together with poly(allylamine), coordinate Gd ions; finally, through the formation of a second polyelectrolyte layer via subsequent reactions with poly(acrylic acid) and poly(allylamine), the 3-(isopropyl-*o*-carboranyl) hydrindone was attached. The average diameter of the obtained NPs was 110 nm, while the zeta potential was about −20 mV. The amount of boron and gadolinium in the final NPs, estimated by laser-induced breakdown spectroscopy, was 0.077 mg/g and 0.632 mg/g, respectively, and the selective toxicity of the nanosystem against cancer cells was demonstrated using different cell lines. In a following work [79], the authors, with the aim of improving the drug release, prepared the same NPs anchoring di(*o*-carborano-1,2-dimethyl)borate to their surface through an ionic bond with the positively charged amino groups of the poly(acrylic acid)/poly(allylamine)polyelectrolyte. According to energy dispersive X-ray spectroscopy, B content in the final NPs was 15.4%, while Gd content was 1.5%, and in vitro tests on liver cancer cells and human fibroblasts evidenced low cytotoxicity of these NPs in a concentration range from 0.05 to 1 mg/mL.

A complex multifunctional nanocomposite simultaneously usable for MRI and photothermal therapy (PTT)-BNCT dual cancer therapy was developed by Icten et al. [80]. In particular, the authors decided to use as a core MnFe_2_O_4_ magnetic NPs, which, compared to iron oxide NPs, display high saturation magnetization and excellent T2-negative contrast [134,135]. MnFe_2_O_4_ NPs were prepared by the solvothermal method and coated with polydopamine, to reduce gold NP and attach boron atoms, introduced using boric acid, through catechol functional groups. The final MnFe_2_O_4_@PDA-Au-BA NPs presented a negative zeta potential of −30.3 mV and Au and B contents of 2.5 × 10^17^ and 3.1 × 10^17^ atoms/mg of sample, respectively, making these NPs appropriate candidates for magnetic targeted BNCT and PTT applications.

### 2.4. Gold Nanoparticles

In recent years gold nanoparticles (AuNPs) have been among the most extensively studied nanomaterials because of their remarkable optical properties related to their plasmon absorption, attracting the interest of researchers in the areas of PTT, biosensing, imaging, and drug delivery [136]. Thanks to these multiple possible applications, AuNPs have also great potential as theranostic agents, solving the problem of locating drugs and allowing the treatment with a single agent [137]. AuNPs can be easily obtained with controlled size and shape and conveniently functionalized [138]; therefore, incorporation of boronated compounds into AuNPs could be a viable approach for the delivery of boron to tumors [139,140]. Importantly, the Au core may be activated during neutron irradiation to form Au-198 releasing β-particles, and the combination of α- and β-particles could possibly elevate the response to BNCT.

Ciani and her group [81] described a bottom-up approach to obtain *o*-carborane functionalized AuNPs covered with poly(ethyleneoxide)-b-poly(caprolactone) diblock-copolymer (PEO-b-PCL) to enhance their hydrophilicity. The AuNPs were prepared through the Turkevich method [141] using sodium citrate as a reducing/capping agent, then were equilibrated with PEO-b-PCL and functionalized with carborane cages (1,2-dicarba-*closo*-dodecaborane(12)-9-thiol, 9SHOCB, or 1,2-dicarba-*closo*-dodecaborane(12)-9,12-dithiol, 9,12SH-OCB) through click chemistry. After optimization of the dodecaborane concentrations and polymer/dodecaborane ratios, the obtained NPs displayed a mean diameter ranging from 35 to 74 nm, depending on the formulation. The boron uptake by rat osteogenic sarcoma cells, measured by neutron autoradiography, ranged from 0.11 to 0.22 ppm and, considering that carboranes with a natural isotopic concentration of ^10^B were employed, the authors assumed that, by using ^10^B-enriched carboranes for functionalization, a factor of 5 could be gained, thus positioning this nanosystem in the therapeutic window (10–30 ppm).

To obtain a tumor-selective boron delivery nanosystem, Wang et al. [82] conjugated gold nanoclusters (AuNCs), prepared following a previously described procedure [142], with the amino derivative of *nido*-carborane ([7- NH_2_(CH_2_)_3_S-7,8-C_2_B_9_H_11_]^-^ (CB), confirming by ^1^H-NMR the formation of an NH_2_-Au bonding. The fluorescence spectra evidenced an enhancement upon the addition of CB to the AuNCs, characterized by an average diameter of 9.4 ± 1.6 nm. The AuNCs-CB were biocompatible with both cancer and normal cells, and by energy dispersive spectrometry the boron content in cancer cells treated with AuNCs-CB resulted higher than the one in control cells receiving only CB (6.98% and 1.96%, respectively). In vivo fluorescence imaging performed on tumor-bearing mice highlighted AuNCs-CB accumulation at the tumor site, demonstrating the applicability of this nanosystem to theranostics, allowing to visualize the boron source delivery to tumors with fluorescent imaging and promoting at the same time the effect of BNCT treatment through imaging-guided therapy.

Theranostic boron-containing AuNPs were developed also by Wu et al. [83], who conjugated commercial citrate-coated AuNPs with PEG moieties, followed by functionalization with boron cage-SH via dodecanethiol azide; ^123^I was also introduced in the AuNPs by click chemistry. Then, to further enhance the tumor retention of these AuNPs, an anti-human epidermal growth factor receptor-2 (HER2) antibody (61 IgG) previously synthesized by the authors, was thiol-modified and grafted to the NP’s surface via a disulfide bridge. The final ^123^I-61-B-AuNPs exhibited an average diameter of 57.59 ± 13.90 nm. The in vitro cellular uptake evaluated by radioactivity measurement in human gastric cancer cells revealed a maximum accumulation of 19.66 ± 2.71% (%uptake/10^6^ cells) and good cell retention. In vivo microSPECT/CT imaging performed on tumor-xenografted mice, followed by ICP-MS quantification, showed that ^123^I-61-B-AuNPs accumulated in the tumor after 12 h with an uptake ratio of 48.32 ± 3.11% ID/mL, higher than the one detected in mice receiving the non-targeted AuNPs (7.43 ± 0.28% ID/mL). Interestingly, the authors hypothesized that the NPs underwent antibody-mediated phagocytosis, since an increased uptake by the thyroid was also observed.

In a following work [84], the same authors developed novel boron and gadolinium containing NPs, potentially useful for combined MRI and BNCT, covered with poly(D, L-lactide-co-glycolide) (PLGA) polymer. As the boronated compound, a boron cage functionalized with an ethylene glycol-SH (BC-EG-SH) chain was selected, after the exclusion of BPA and BPA-fr, which led to lower EE values. These PLGA-Gd/B-AuNPs were prepared by emulsification and centrifugation of a mixture obtained by adding PLGA and BC-EG-SH dissolved in acetone to an aqueous solution of gadopentetate dimeglumine and commercial citrate-coated AuNPs. The obtained NPs exhibited an average diameter of 172.8 ± 68.5 nm, and TEM micrographs confirmed that PLGA encapsulated the AuNPs; the boron EE, determined through ICP-MS, was 31.1 ± 2.5%. In vitro tests on human gastric cancer cells showed that the accumulation of PLGA-Gd/B-AuNPs increased with time, reaching a maximum of 0.68 ± 0.24 µg B/10^7^ cells 36 h after incubation, and was higher than the accumulation of BC-EG-SH alone, highlighting that the nanoassembly might increase boron delivery to the tumor site. The PLGA-Gd/B-AuNPs were retained at the periphery of larger xenografts of tumor-bearing mice, while penetrating deeper into the smaller ones, with a boron content of 24.25 ± 5.05 and 76.55 ± 11.75 µg/g sample, respectively, determined by ICP-MS.

Feiner and her group [85] proposed a pretargeting strategy involving the preliminary administration of trans-cyclooctene (TCO) functionalized trastuzumab followed by the in vivo click reaction with tetrazine (Tz)-functionalized boron-rich AuNPs. To functionalize trastuzumab, commercially available TCO-NHS was conjugated to the amino groups of the mAb lysine residues, obtaining on average two TCO per molecule as determined by photometric titration. PEG-stabilized AuNPs were prepared by adding sodium borohydride to an aqueous solution of chloroauric acid, CuCl_2_, and NH_2_-PEG5k-SH; then, cobalt bis[dicarbollide] (COSAN) and Tz-PEG5k-NHS were grafted on the NP surface by incubation. After functionalization with COSAN and Tz derivatives, the average diameter of the obtained NPs increased from 27.3 ± 4.0 nm to 39.6 ± 0.8 nm and the zeta potential turned from positive (7.9 ± 9.4 mV) to negative (−33.6 ± 3.82 mV) values. The NP composition was investigated through ICP-MS, revealing high boron content (195 µg/mg). The occurrence of the click reaction between the TCO-mAb and the Tz-functionalized AuNPs was proved using agarose gel electrophoresis, while the biocompatibility of this system was confirmed by in vitro testing on breast cancer cells. Finally, to enable in vivo tracking of the AuNPs by positron emission tomography (PET), the AuNP core was doped with ^64^Cu; however, PET-computed tomography (PET-CT) scans of tumor-bearing mice receiving the ^64^Cu-radiolabeled AuNP evidenced a maximum tumor uptake not significantly different from the controls (receiving trastuzumab without TCO functionalization or AuNPs, without any pretargeting). To explain this lack of significance, the authors performed further in vitro internalization studies, leading to the hypothesis that the click reaction with trastuzumab available on the cell surface and the direct internalization of the AuNPs are in competition, with the second one possibly being the main driving force for tumor retention.

Human serum albumin (HSA) and AuNPs feature a synergy resulting in reduced interaction with other plasma proteins, increased NP stability, passive and active targeting of the conjugates to malignant cells leading to increased selectivity, and enhanced translocation of the NPs into the brain [143]. Therefore, Popova et al. [86] developed AuNPs functionalized with a undecahydro-*closo*-dodecaborate-HSA (B-HSA) conjugate, synthesized by click chemistry and adapting a procedure described in the literature [144]. Through an S-Au bond involving the only cysteine residue of HSA free from disulfide bridges (Cys34), B-HSA was conjugated to citrate-coated AuNPs, prepared using the citrate method [145], obtaining B-HSA-AuNPs with a diameter of 28.93 ± 0.08 nm, negative zeta potential (−22.43 ± 1.12 mV), and boron content of 0.307 µg B/mg of gold, determined by ICP-OES. The B-HSA-AuNPs exhibited low cytotoxicity on human mammary adenocarcinoma cells.

### 2.5. Silica Nanoparticles

Silica nanoparticles (SiNPs) are frequently selected for biomedical applications due to their low toxicity and easy workup in a wide range of sizes and morphologies. In particular, their potential use as boron carriers for BNCT has been investigated by several research groups [146,147,148,149]. A common issue arising from the development of elemental boron NPs might be the irregular shape and the broad size distribution of the boron NPs obtained from commercial boron powders through milling techniques [150], and this limitation can be overcome by encapsulating boron NPs into a silica shell. For example, Walton et al. [87] prepared boron NPs by milling boron powder in hexane in the presence of 10-undecenoic acid (UNDCA), whose terminal alkene group was involved in the following hydrosilylation reaction for silica coating, carried out using triethoxysilane and tetraethoxysilane. By TEM analysis, the authors showed that, contrarily to the UNDCA-capped BNPs, the silica-covered BNPs presented a spherical shape and monodisperse distribution.

With the aim of obtaining a nanosystem potentially useful in MRI-guided BNCT, Kuthala et al. [88] incorporated Gd(III)-diethylenetriamine penta-acetic acid (DTPA) complexes onto the surface of ^10^B NPs surrounded by a silica layer, to achieve good biocompatibility. Moreover, to add tumor-targeting properties, amino functions introduced on the NP surface were exploited for conjugation with FITC-labeled RGD-K peptides through amidic bond formation, using EDC to activate the peptide carboxylic groups. The so obtained ^10^BSGRF NPs were characterized by an average diameter of about 90 nm and positive zeta potential (about 14.8 mV); the ^10^B/Gd ratio, determined by ICP-MS, was about 4.06. Confocal laser scanning optical microscopy observation and flow cytometry analysis evidenced a significant uptake of ^10^BSGRF NPs in glioma cells and, after exposure to neutron irradiation, a drop in cell viability. MRI performed on tumor-bearing mice receiving ^10^BSGRF NP showed appreciable signals at the tumor site, with ^10^B accumulation of about 50.5 μg/g after 24 h, determined through ICP-MS, associated with good ^10^B retention within the tumor, and rapid clearance from the blood. Thermal neutron irradiation of mice receiving ^10^BSGRF NPs resulted in reduced tumor growth and prolonged survival, compared to the controls (from 22 to 39 days).

A particular type of silica nanodispersed material is represented by mesoporous silica NPs (MSN), characterized by pores of tunable diameter and large volume for efficient drug loading, accompanied by high surface area [151,152]. Lai et al. [89] described the preparation of MSN incorporating large amounts of *o*-carborane and functionalized with amino groups for conjugation with amino-galactoside ligands, for targeting hepatic cancer, and with the Cy3 dye for fluorescent imaging. The MSN were prepared from tetraethylorthosilicate added to an aqueous solution of hexadecyltrimethylammonium bromide mixed with NaOH and ethanol, and then amino-functionalized using APTMS. After reacting with trimethylchlorosilane to graft trimethylsilane groups onto the mesopore surfaces, Cy3 was incorporated into the MSNs by a competition method, and a mixture of galactosyl amino ligands was conjugated by incubation, obtaining two formulations decorated with two different galactosides, M-Gal-Cy3@MSN and T-Gal-Cy3@MSN. The amount of galactoside ligands on the NP surface was estimated through an anthrone-sulfuric acid colorimetric assay (262.74 ± 14.12 and 289.73 ± 13.19 nmol/mg for M-Gal-Cy3@MSN and T-Gal-Cy3@MSN, respectively). The NPs were filled with *o*-carboranes by impregnation using dichloromethane as the solvent, resulting in 539 ± 0.066 µg and 445 ± 0.049 µg of boron per mg of M-Gal-Cy3@MSN and T-Gal-Cy3@MSN, respectively, as determined by ICP-MS and confirmed by thermogravimetric analysis and differential thermal analysis. Confocal imaging and ICP-MS showed the internalization of Gal-Cy3@MSN in HepG2, hepatic cancer cells, with more than 10^11^ boron atoms accumulated in each cell, which is significantly higher than the accumulation obtained in cells treated with BSH at the same conditions (2.76 × 10^9^). The efficacy of this novel BNCT system was assessed by evidencing reduced colony formation ability of the HepG2 cells receiving thermal neutron irradiation after treatment with T-Gal-B-Cy3@MSN.

Chondrosarcoma-targeting, BSH-loaded MSN were developed by Vares et al. [90]. Using APTES, the MSN were functionalized with amino groups that formed amide bonds with bromoacetic acid, the conjugation with BSH through a nucleophilic attack to follow; then, a cell-penetrating peptide (ACPP) synthesized by the authors was conjugated to BSH through the reaction between SH and amino groups; a PEG layer was also added to improve biocompatibility and blood circulation time, and FITC labeling and Gd-DTPA grafting were carried out for in vitro and in vivo tracking. DLS analysis showed an average diameter of 200 nm and a negative zeta potential (lower than −25 mV); the boron content, measured by ICP-MS, was 5 × 10^17 10^B atoms per mg NPs. After confirming the tumor uptake of boronated MSN in chondrosarcoma-bearing mice by MRI, in vitro studies on CH-2879 and on an ALDH+ radioresistant cancer stem-like cell subpopulation exposed to neutron beam irradiation evidenced that, even if apoptosis induction after 24 h was limited, significant DNA damage and lower clonogenic survival occurred. Interestingly, the authors also highlighted that cancer stem cells, which are more resistant to conventional X-ray therapies, did not show any significant difference from CH-2879 cells upon BNCT treatment.

To target integrin receptors overexpressed in pancreatic tumors, Wang and his group [91] designed a complex system composed of a hydrophobic carborane core (CB) covalently conjugated to dendritic mesoporous silica nanospheres (DMSNs), synthesized via an oil–water biphasic stratification approach, loaded with doxorubicin and coated with a hydrophilic cRGD-modified polyethyleneimine (cRGD-PEI) polymer, responsible of the integrin-targeting activity. The so-called DOX/CB@DMSNs exhibited an average diameter of 136 nm and a zeta potential of +46.3 mV, due to the numerous PEI amino groups, confirming the effective cRGD-PEI covering, also assessed by FTIR spectroscopy. The doxorubicin loading, measured by UV–visible spectroscopy following a not clearly described procedure, was 84.3 mg/g, while ICP-MS revealed a boron loading of 141.5 mg/g. In vitro studies performed on human pancreatic carcinoma cells showed no DOX/CB@DMSNs toxicity and their cellular uptake was observed by confocal laser scanning microscopy. Finally, in vivo biodistribution experiments conducted by ICP-MS on tumor-bearing mice evidenced a high tumor-to-blood ratio (about 27.1) 16 h after DOX/CB@DMSNs injection, corresponding to 24.4 µg ^10^B/g, which is sufficient for BNCT applications.

**Figure 1:**
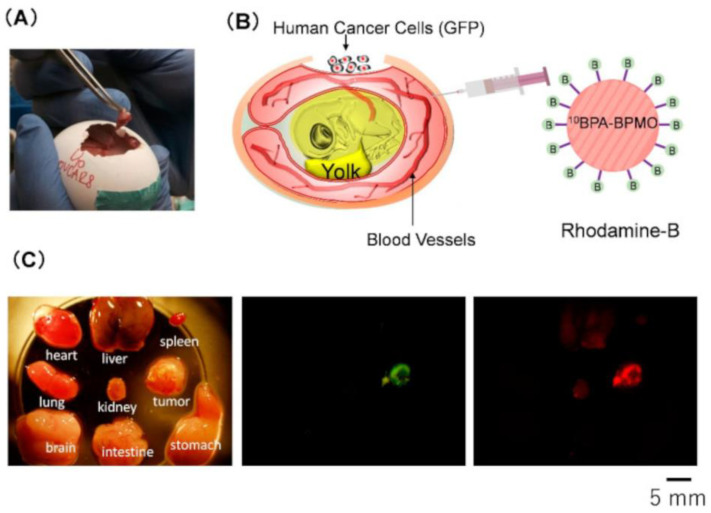
(**A**) Tumor formation on the CAM by transplanting cancer cells; (**B**) a CAM-based assay to examine tumor accumulation of nanoparticles. The tumor is formed using green fluorescence protein (GFP) expressing cells; (**C**) detection of ^10^BPA-BPMO in tumor. Reproduced from [92] under the Creative Commons Attribution (CC BY) license (http://creativecommons.org/licenses/by/4.0/). 26 September 2022.

A peculiar kind of MSN is represented by biodegradable periodic mesoporous organosilica (BPMO) NPs, containing biodegradable chemical bonds (e.g., di- and tetrasulfide bonds) within the framework of the particle, designed to undergo degradation and consequent active release under specific conditions such as redox and low pH environments [153,154]. In a study from Tamanoi et al. [92], diol-modified BPMO NPs were synthesized using bis [3-(triethoxysilyl)propyl]tetrasulfide and 1,2-bis(triethoxysilyl)ethane as precursors and, to increase the dispersibility, phosphonate surface modification was performed; then, through a procedure leading to the opening of the 3-glycidyloxypropyl trimethoxysilane epoxide ring, a diol group was introduced for BPA binding. The NPs showed an average diameter of 170 nm and zeta potential of −42.48 mV; the boron content, determined by ICP-OES, was 25% of the NP weight. Noteworthy, to assess BPMO NPs uptake, investigated by confocal microscopy and flow cytometry in different cancer cell lines, the authors employed the CAM (chorioallantoic membrane) model, which implies the use of fertilized eggs grafted with human ovarian cancer cells expressing the green fluorescent protein. Confocal microscopy evidenced the superimposition of the green and red fluorescence deriving from cancer cells and from the Rhodamine B-labeled BPMO NPs, respectively, and after thermal neutron irradiation, significant tumor growth inhibition was observed (Figure 1).

In a recent work, Sharma and his group [93] developed multimodal boron vehiculating silica-coated Cr-doped zinc gallate-based persistent luminescence NPs (PLNPs), by exploiting the reaction of boric acid with the vicinal diol functions present on the NPs surface, introduced by sequential surface functionalization with APTMS and dicarboxy-terminated polybutadiene followed by reduction with KMnO_4_. To achieve tumor targeting, these NPs were functionalized with a pH-(low)-insertion peptide, exploiting the extracellular acidity typical of several diseases, including tumors [155]. This complex nanosystem possessed tumor-homing capacity in fibrosarcoma-bearing mice observed under a photon imager, and BNCT efficacy was proved by measuring the tumor growth in melanoma-bearing mice undergoing thermal neutron irradiation after PLNPs injection.

### 2.6. Boron Carbide Nanoparticles and Quantum Dots

Boron carbide (B_4_C), endowed with high neutron absorptivity, has been studied as a possible material for BNCT agents [156]. For example, Mortensen et al. [94] evaluated the potential use of B_4_C NPs in T cell-guided BNCT, and, to improve cellular adhesion and uptake, functionalized their B_4_C NPs with the transactivator of transcription (TAT) peptide, proving their efficacy as BNCT agents in a murine melanoma model [95].

More recently, Tsuji and his group [96] developed B_4_C NPs, prepared by pulsed laser melting in ethanol and successively covered with poly-L-lysine and poly-γ-glutamic acid, and functionalized them with iron-loaded transferrin to obtain a cancer-targeting nanosystem potentially useful in BNCT. The internalization of FITC-labeled, transferrin-functionalized B_4_C NPs was shown by confocal images in HeLa cells overexpressing the transferrin receptor and confirmed by TEM in mouse ex vivo tumor tissues.

Based on the idea that immune phagocytic cells may represent a promising method for the direct delivery of drugs to tumors, Kozien et al. [97] studied the interaction between IgG-functionalized B_4_C NPs, prepared from B_4_C powder obtained by the direct reaction between boron and soot under argon flow, and two cell lines, MC38 murine colon carcinoma and RAW 264.7 mouse macrophages cell line. Flow cytometry analysis confirmed the higher uptake of the fluorescently labeled NPs by macrophages than by cancer cells.

Singh et al. [98] proposed a simple solvothermal method for producing B_4_C quantum dots at relatively low temperature. B_4_C was synthesized from boron tribromide and carbon tetrachloride using magnesium turnings as the reducing agent, resulting in the formation of 7 nm diameter dots with hexagonal phase, as observed from XRD. The authors confirmed the absence of toxicity on different human cell lines, while a cytotoxic effect resulted in HeLa cells treated with the B_4_C quantum dots and thermal neutron irradiated.

The already mentioned pretargeting system involving TCO-modified trastuzumab and its click reaction with a Tz-modified boron-vehiculating nanosystem, firstly developed by Feiner et al. [85] using AuNPs, was also applied to boron-doped carbon dots (BDDs) functionalized with Tz moieties [99]. Indeed, the small size of carbon dots might be beneficial in a pretargeting strategy, because of the fast elimination from circulation and tissues. The final BDDs-Tz exhibited a size of 6.7 ± 1.8 nm, a zeta potential of −2.7 ± 0.3 mV, as measured by atomic force microscopy, and a boron content of 3.4 ± 0.3%, determined by ICP-MS. Internalization studies in human breast cancer cells using [^18^F]BDDs-Tz showed 0.12 ± 0.02% internalized particles. In vivo biodistribution studies were conducted by PET imaging on tumor-bearing mice receiving the pretargeting treatment with TCO-trastuzumab followed by [^18^F]BDDs-Tz, highlighting the retention of the boronated dots in the tumor tissue.

### 2.7. Polymeric Nanoparticles and Micelles

Block copolymers such as PEG-block-poly(D,L-lactide) (PEG-b-PLA) or PEG-block-poly(D,L-lactide-co-glicolide) (PEG-b-PLGA) have been frequently used as constituents of spontaneously formed polymeric NPs. In fact, their core can accommodate hydrophobic drugs to be control-released, while the outer hydrophilic PEG corona protects the NPs from immune surveillance [157]. These NPs also showed other favorable features such as high biocompatibility, long blood circulation time, and high biodegradability [158]. However, their use in BNCT applications is hampered by the leakage of core-incorporated hydrophobic compounds within the bloodstream, due to the interaction of the NPs with some blood components [159].

This problem was encountered by Meher et al. [100], who developed carborane-loaded PEG-b-PLGA NPs for targeted BNCT and PET imaging of prostate cancer. PEG-b-PLGA was linked through an amidic bond to 2-(3-((S)-5-amino-1-carboxypentyl)ureido) pentanedioic acid (ACUPA), a small-molecule ligand targeting prostate-specific membrane antigen (PSMA) [160], and to the radiometal chelator deferoxamine B (DFB) exploiting the formation of a thioureidic function. The so-obtained functionalized polymers were mixed in 25:25 or 25:75 ratios in the presence of carborane and, after a process of nanoemulsification, the obtained NPs, displaying by ICP-OES a boron incorporation of 1.86 wt%, were radiolabeled with ^89^Zr. DLS measurements evidenced an average diameter ranging from 150 to 165 nm, while the zeta potential decreased by increasing the percentage of DFB in the formulation (from -31.0 ± 1.16 mV to -37.0 ± 0.71 mV). Binding, internalization, competition radioligand binding, and boron uptake of the NPs were observed on PC3-pip cells, overexpressing PSMA, but not on PC3-flu cells, characterized by low PSMA expression. However, the PSMA-targeted NPs did not show increased tumor accumulation in PC3-pip xenografted mice, with consequent low boron levels in the tumor tissue, probably due to the rapid carborane release in the serum.

To overcome the issue of hydrophobic compound leakage, Sumitani et al. [101,102] decided to covalently link the boronated compound to the polymers that constitute the NPs. Indeed, these authors prepared micelles via radical polymerization of PEG-b-PLA, containing an acetal group at the PEG end and a methacryloyl group at the PLA end, using polymerizable carborane (VB-carborane) as a cross-linker, thus obtaining cross-linked (CL) micelles with an average diameter of 85.3 nm and almost neutral zeta potential (−0.69 ± 0.33). ^1^H-NMR confirmed the VB-carborane covalent binding to the micelle core, with a boron content of 0.26 wt% (loading efficiency: 3.1%), determined by ICP-OES. Through fluorescence intensity measurements, almost no leakage of VB-carborane from CL micelles was observed, contrary to the non-cross-linked (NCL) ones prepared under the same conditions; in accordance with these data, significantly lower cytotoxicity of CL compared to NCL micelles was observed on colon carcinoma cells. The biodistribution of ^125^I-labeled CL and NCL micelles in tumor-bearing mice highlighted, 24 h after injection, prolonged blood circulation time (7.9% ID/g) and higher tumor accumulation (2.9% ID/g) of the CL micelles compared to the NCL ones (3.1% ID/g in blood, 1.8% ID/g in tumor).

A different strategy to suppress the boron leakage from polymeric NPs was proposed by Xiong et al. [103], who developed doxorubicin-loaded polymeric NPs composed of the self-assembling carborane-conjugate poly(ethylene glycol)-b-poly(L-lactide-co-2-methyl-2(2-dicarba-*closo*-dodecarborane)propyloxy-carbonylpropyne carbonate) (DOX@PLMB). The obtained NPs, with an average diameter of 107.8 nm, showed a drug loading of 9.9 ± 1.2 wt% and an EE of 60 ± 7.6%, as determined by UV–visible spectrometry; moreover, low carborane leakage was observed using dialysis and ICP-OES. DOX@PLMB pharmacokinetics was investigated in vivo in both healthy and tumor-bearing mice, revealing, in the latter group, prolonged boron presence in the blood, altogether with tumor accumulation, as also confirmed by fluorescent images of resected tissues. Tumor growth inhibition and suppression were observed in tumor-bearing mice injected with DOX@PLMB followed by thermal neutron irradiation.

Moreover, Chen and his group [104] aimed at exploiting the combinatory antitumor efficacy of radiotherapy with doxorubicin, which is known to enhance tumor cell radiosensitivity [161]. In this work, a polymer composed of the tumor-penetrating iRGD peptide, PEG, and polycarboxycaprolactone (PCCL) was covalently linked to BSH and used to prepare polymeric NPs, encapsulating doxorubicin in the hydrophobic core by solvent evaporation technique. NPs of 24.97 ± 3.66 nm size and −15.50 ± 1.70 mV zeta potential were obtained, with 3.81% boron loading, evaluated by ICP-MS, and 2.30% doxorubicin loading, estimated by UV analysis. Boron in vitro uptake by human lung cancer and mouse melanoma cells incubated with the iRGD-PEG-PCCL-BSH NPs resulted in 21.90 ng ^10^B/10^6^ cells, measured by ICP-MS, which was also used to evaluate the in vivo boron distribution in tumor-bearing mice, obtaining a tumor:blood ratio of 14.11.

Gao and his group [105] circumvented boron leakage from polymeric NPs by covalently binding BSH to the side chain of poly(chloromethylstyrene) (PCMS) segments in the PEG-b-PCMS prepolymer, obtaining the polyanion PEG-b-poly((*closo*-dodecaboranyl)thiomethylstyrene). Boron cluster-containing redox NPs (BNPs) were formed from the aforementioned polyanion and the polycation PEG-b-poly(4-(2,2,6,6-tetramethylpiperidine-N-oxyl)aminomethylstyrene), exhibiting an average diameter of about 36 nm and zeta potential close to zero. Uptake studies performed on tumor and healthy cells evidenced that BNPs uptake was significantly higher than that of BSH, but lower than BPA, used as controls. However, the authors stated that these NPs were still promising because the delivery of these compounds to the tumor environment is one of the important factors for practical treatment in vivo. Indeed, an in vivo pharmacokinetic study on tumor-bearing mice showed that BNPs are endowed with increased accumulation and prolonged and selective retention in the tumor environment, conditions required to improve the therapeutic effect of thermal neutron treatment; indeed, after thermal neutron irradiation, significant tumor progression inhibition was observed, compared to the controls. Moreover, the presence of a ROS scavenging function in the BNPs might cause the suppression of adverse effects related to the increase of leukocyte recruitment, since mice undergoing neutron irradiation after BNPs administration presented leukocyte levels not significantly different from untreated mice.

Another interesting study, reporting the comparison between boron-loaded NPs composed of PLGA or poly(L-lactide-co-glycolide) (PLLGA), was reported by Takeuchi and coworkers [106], based on the observation that PLLGA NPs induced decreased initial burst of the hydrophobic drug compared to PLGA NPs [162]. *O*-carborane-loaded PLGA and PLLGA NPs were prepared by the emulsion solvent evaporation method, leading to the formation of 100 nm or 150 nm diameter NPs, with *o*-carborane contents ranging from 4.8 to 5.6%, determined by ICP-OES. Using dialysis and ICP-OES, the two formulations were compared for the in vitro *o*-carborane release, which resulted significantly lower for the PLLGA ones. Accordingly, in an in vivo biodistribution study on melanoma-bearing mice the maximum boron concentration value in the tumor (113.9 ± 15.8 µg/g of tissue), determined by ICP-OES, was obtained with 100-nm PLLGA NPs.

In a following work [107], the same authors investigated the effects of chitosan (CS) coating on *o*-carborane-loaded PLGA NPs, with apparently no clear explanation on the choice of using PLGA rather than PLLGA, seeing the previously reported results. Due to its favorable safety profile, CS has been used in several studies as the component of boron-vehiculating NPs for BNCT application [163]. This coating simply exploited the charge interaction between the negative surface of the NPs and the CS positive charges, and the coated PLGA-NPs exhibited an average diameter of 113.6 ± 32.5 nm, comparable to the diameter of bare PLGA NPs. The *o*-carborane content of CS-coated NPs, determined by ICP-OES, was 5.18 ± 0.16%, and the particle surface charge number density, calculated on the basis of the electrophoretic mobility, was 20.8 mM, compared to the value of −1.91 mM obtained for bare NPs: the turning from negative to positive charge confirmed the chitosan coating. The *o*-carborane leakage in vitro was significantly lower from CS-coated NPs as compared to the bare ones, and confocal laser scanning microscopy and FACS analysis evidenced higher melanoma cell internalization of CS-coated NPs, with a number of boron atoms per cell 1.8 times higher than the one vehiculated by bare NPs.

Considering that boronic acid derivatives can selectively bind to sialic acid receptors expressed on tumor cells membranes [164,165,166], Soleimanbeigi et al. [108] synthesized thermo-responsive NPs composed of chitosan-poly(N-isopropylacrylamide) conjugated with BPA through amide bonds between the BPA carboxylic group and the CS amino groups; moreover, to increase the potential anticancer efficacy of the system, BPA was also loaded in the NP core. The efficiency of BPA conjugation was confirmed by ^11^B NMR, and the obtained NPs showed an average diameter of 145.39 ± 1.5 nm and negative zeta potential (–20.2 ± 0.8 mV); the declared BPA EE, measured by HPLC, was >95%. Through UV–visible spectrometry, increased release of BPA when increasing the temperature from 37 to 40 °C was assessed. For the in vitro cellular uptake studies, the same NPs encapsulating fluorescent curcumin were prepared, and flow cytometry evidenced higher cellular uptake in the case of cells receiving BPA-functionalized NPs compared to the control cells, treated with non-targeted NPs.

**Figure 2:**
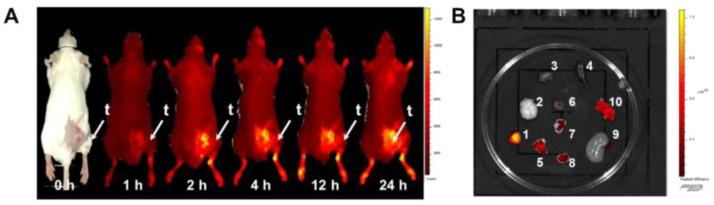
Fluorescence imaging showed accumulation of BPN in the tumor region. (**A**) Fluorescence images of 4T1 tumor-bearing mice 0, 1, 2, 4, 12, and 24 h post intravenous injection of 1 mg of BPN. (**B**) Fluorescence images of variant organs in 4T1 bearing mice 24 h post injection of BPN (TBPP = 1 mg): 1: tumor, 2: brain, 3: kidney, 4: spleen, 5: fat, 6: heart, 7: lung, 8: muscle, 9: liver, and 10: large intestine. Reproduced with permission from Shi et al. [109], 2018.

**Figure 3:**
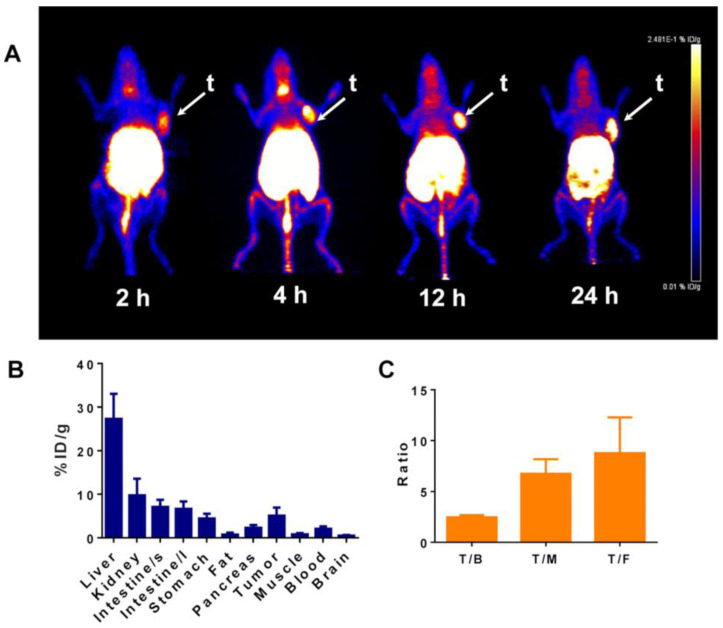
(**A**) Whole-body maximum intensity projection PET images showing the uptake of BPN. Tumor (t) was indicated by white arrows. (**B**) Corresponding biodistribution of ^64^Cu−BPN in B16−F10 bearing mice 24 h post injection. Data are means ± SD (n = 4 mice). (**C**) Tumor-to-blood (T/B), tumor-to-muscle (T/M), and tumor-to-fat (T/F) of % ID/g ratios. Reproduced with permission from Shi et al. [109], 2018.

Boronated porphyrins are promising compounds because of their high accumulation and long retention in tumors [167], their fluorescent properties, and their possible use to combine BNCT and photodynamic therapy. Therefore, Shi et al. [109] proposed a novel boronated porphyrin-loaded poly(lactide-co-glycolide)-monomethoxy-poly(ethylene-glycol) (PLGA−mPEG) nanocomplex (BPN), consisting in PLGA-mPEG micelles incorporating a tetraboronated porphyrin obtained by the dialysis method, which might be employed as a theranostic agent for both PET imaging and BNCT. The micelles had an average diameter of about 100 nm and zeta potential of −39 mV, and the success of porphyrin encapsulation was assessed by IR analysis; consistent uptake in the cytoplasm and nucleus of cancer cells was observed by confocal microscopy and ICP-OES. Exploiting optical imaging, BPN tumor accumulation was also observed in tumor-bearing mice and then, using a micro-PET system, the biodistribution and the pharmacokinetics of [^64^Cu]-BPN were monitored (Figure 2 and Figure 3). By optimizing the administration protocol, the authors obtained a tumor boron concentration of 130 ppm, determined by ICP-OES, with a tumor:blood ratio of 33.85 ± 5.73, and thermal neutron irradiation of tumor-bearing mice injected with BPN resulted in tumor growth suppression and prolonged animal survival.

**Figure 4:**
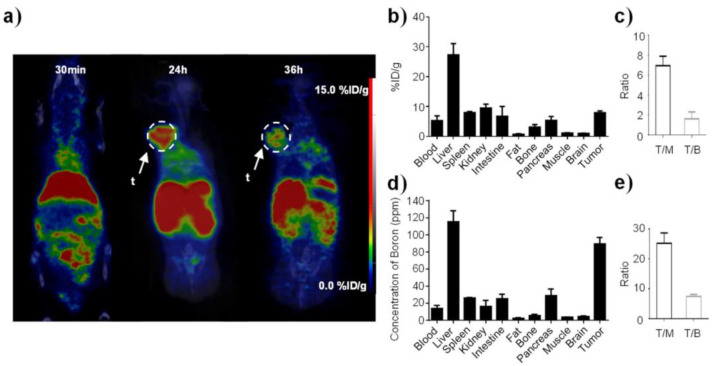
In vivo biodistribution of ^64^Cu-DSPE-BCOP-5T in 4T1 tumor-bearing mice. (**a**) Representative whole-body coronal PET/CT fusion image of ^64^Cu-DSPE-BCOP-5T at different times postinjection. The tumor (t) is indicated by the white arrow (n = 3). (**b**) Ex vivo biodistribution of ^64^Cu-DSPE-COP-5T in 4T1 tumor-bearing mice 24 h postinjection (mean ± SE, % ID/g) (n = 6). (**c**) Tumor-to-muscle (T/M) and tumor-to-blood (T/B) ratios of ^64^Cu-DSPE-COP-5T. (**d**) Boron concentrations in the indicated organs after three separate injections of DSPE-BCOP-5T (mean ± SE, μg/g) (n = 6). (**e**) Tumor-to-muscle (T/M) and tumor-to-blood (T/B) ratios of boron concentrations. Reproduced with permission from Shi et al. [110], 2020.

Following this work, the same group [110] described the synthesis of a carborane-conjugated polymer named BCOP-5T, obtained from the polymerization of 5,10,15,20-tetrakis(4-aminophenyl)porphyrin (TAPP) and DF5T (a pentathienyl aldehydic derivative) in the presence of carborane, and then coupled it to DSPE-PEG and obtained self-assembling water-dispersible NPs. The boron content in BCOP-5T, measured by ICP-OES, was 11.38 ± 0.47%, and the DSPE-BCOP-5T NPs had a diameter of 110.1 ± 10.9 nm and zeta potential of −22.4 ± 0.6 mV. The NP uptake in tumor cells was studied following the fluorescence signal derived from TAPP by confocal microscopy, and quantifying the cellular boron content by ICP-OES, which reached 300 ppm in 24 h. Incubation of cancer cells with DSPE-BCOP-5T NPs, followed by neutron irradiation, induced apoptosis in approximately 54% of the cell population. Micro-PET/CT was used to investigate the in vivo biodistribution of ^64^Cu-labeled DSPE-BCOP-5T NPs in tumor-bearing mice, evidencing tumor accumulation, also confirmed by ICP-OES analysis on excised tissue (Figure 4). After thermal neutron irradiation, the tumor growth was suppressed and the animals benefited from prolonged survival.

To develop a BNCT agent targeting hepatocarcinoma cells, Zhang and his group [111] developed a PEGylated galactose polymer containing carborane clusters. The amphiphilic polymer mPEG-b-poly(lactic acid-co-5-methyl-5-propargyloxycarbonyl-1,3-dioxan-2-one (mPEG-b-P(LA-co-MPC)) was synthesized by ring-opening copolymerization of mPEG-initiated cyclic carbonate monomer and lactide, followed by the introduction of carborane clusters and then galactose into the polymer, by click reaction. The boron content in each polymer chain was found to be about 3 wt% by ICP-MS, and the deriving self-assembled micelle diameter was 135 nm. By confocal laser scanning microscopy, cellular uptake was observed in HepG2 cancer cells and, after thermal neutron irradiation, an increased apoptosis rate was detected.

The combination of arene ruthenium(II) complexes and carboranes has unexplored potential in medicine, since their applications are hampered by the hydrophobicity of such complexes. Therefore, to exploit the chemistry of carborane-containing arene ruthenium complexes in aqueous environment, Romero-Canelón and coworkers [112] developed Pluronic^®^ micelles encapsulating the complex [Ru(p-cymene) (1,2-dicarba-*closo*-dodecarborane-1,2-dithiolate)]. The encapsulation was achieved by adding a tetrahydrofuran solution of the carborane-containing complex to an aqueous solution of the amphiphilic triblock copolymer P123 [168]. The micelles, exhibiting a diameter of about 19 nm, were tested in vitro on A2780 and cisplatin-resistant A2780cisR human ovarian cancer cells, causing, as resulted from ICP-MS, higher accumulation of the boronated compound in cells receiving the carborane-loaded micelles than in control cells, treated with the carborane-containing arene complex alone; this result correlated with higher antiproliferative effect after thermal neutron irradiation.

The first example of polymeric nanostructures based on [B_12_H_12_]^2-^ ion pairing with poly(ethylene oxide)-block-poly(2-(N,N,N′,N′-tetramethylguanidium)ethyl acrylate) (PEO-b-PGEA) block copolymers, forming by co-assembly of guanidinium moieties with [B_12_H_12_]^2-^, was presented by Li’s group [113]. The authors, by varying the block length, manipulated the size and shape of the NPs, obtaining worm-like, rod-like, and spherical boron NPs. Interestingly, flow cytometry studies demonstrated that the rod-like NPs exhibited the highest uptake in glioblastoma cells and, besides the shape-effect, this might be explained considering that the formation of rod-like NPs required the use of the copolymer with the longest polycationic block, having the highest fraction of guanidine moieties, possibly enhancing the membrane crossing by the so-called “arginine magic” phenomenon [169].

### 2.8. Other Nanoparticles

Hwang et al. [114] developed a novel potential BNCT nanosystem composed of a boron and cobalt oxide core grafted with carbon NPs functionalized with poly(acrylic acid), to achieve water dispersibility and the conjugation of H_2_N-PEG-folate for cancer targeting. With ICP-OES, the atomic ratio among B:Co:C (1:1.54:10) in the final folate-grafted BCo@CNPs was estimated. Confocal optical images of HeLa cells displayed the internalization of the folate-grafted BCo@CNPs, and the BNCT efficacy of this nanosystem was confirmed by a significant drop in cell viability after thermal neutron irradiation.

Folic acid functionalization was exploited also by Achilli and his group [115] to enhance BPO_4_ NPs selectivity towards tumor cells, thus limiting undesirable cytotoxic effects on blood cells. BPO_4_ was selected as an inexpensive standard material and was prepared in NP form through a top-down method by grounding followed by several wet grinding cycles. The NPs surface was covered with a silica shell and amino groups, introduced using APTES, were exploited for conjugation with folic acid. The BPO_4_-folate NPs had an average diameter of about 255 nm, a zeta potential of −19 mV, and the boron and phosphorous contents, determined by ICP-OES, were 9.8 and 26.3 wt%. BPO_4_-folate NPs exerted no hemolytic effect on erythrocytes and did not induce platelet aggregation, contrarily to non-functionalized BPO_4_ NPs.

Considering the high time and cost consumption of the ^10^B-enrichment process, limiting the clinical advances of BNCT agents, some research groups focused on the development of boron NPs whose efficacy only depends on the ^10^B natural abundance. In this context, Chiang et al. [116] developed polymer-coated boron carbon oxynitride (BCNO) NPs, possessing inherent luminescence, as a potential theranostic agent for BNCT. The NPs, prepared by a low-temperature sintering process using boric acid as a boron source, were coated with PEI or PEG. The particle average diameter was 5.1 ± 0.67 nm, while the zeta potential was positive (about +29 mV) in the case of PEI-coated NPs, and negative (about −29 mV) for PEG-coated NPs; the nanoformulations were analyzed by ICP-OES, revealing about 30% of boron atoms per NPs. By MTT assay on astrocytoma cells, polymer-coated BCNO NPs evidenced lower cytotoxicity than bare ones; PEI-coated NPs showed higher internalization, as determined by fluorescence microscopy, flow cytometry, and ICP-MS, with about 48 µg of boron/g of cells. Moreover, PEG- and PEI-coated BCNO NPs exhibited effective tumoricidal properties on astrocytoma cells undergoing thermal neutron irradiation, due to the high boron content and the enhanced intercellular localization achieved through polymer functionalization.

Another material that is gaining increasing interest in the biomedical field is detonation nanodiamonds (DNDs), featuring high biocompatibility, low toxicity, and tunable surface functionalities [170]. Nishikawa and his group [117] prepared polyglycerol-coated DNDs conjugated to phenylboronic acid, able to exert tumor growth suppression effect in tumor-implanted mice after thermal neutron irradiation.

### 2.9. Exosomes and Biomimetic Vesicles

Extracellular vesicles (EVs) are membrane-derived nano- and microvesicles secreted by all cell types, fulfilling both physiologic and pathologic functions [171]. Among the different types of EVs, exosomes are endowed with several characteristics suitable for drug delivery, being nano-sized, biocompatible, non-toxic, scarcely immunogenic, and possessing targeting ability and organotropism [172].

The homing ability of exosomes, along with their capacity of crossing the blood–brain barrier, led to the study of this kind of vehicle also in the field of BNCT. Li and his group [118] used exosomes derived from the macrophage line RAW 264.7, as nanocarriers for BCDs, which were obtained by the hydrothermal method from BPA and glucose, and exhibited a boron content of 0.78 ± 0.06%, measured by ICP-MS. Exosomes were obtained by sequential centrifugations, and encapsulation was achieved by incubating the optimal 1:2 (*w*/*w*) ratio of BCDs and exosomes at 37 °C, obtaining hollow structures with an average diameter of 96.91 ± 1.26 nm and zeta potential of −15.14 ± 3.19 mV; a drug loading of 17.90 ± 0.27% and an EE of 85.24 ± 1.12% were indirectly determined by UV–visible spectroscopy. By fluorescent cell imaging, the authors demonstrated the uptake of BCDs-exosomes in glioma cells and, according to the ICP-MS data, the amount of uptaken boron was sufficient for BNCT applications. In vivo biodistribution studies in mice evidenced BCDs-exosome accumulation in the brain, and thermal neutron irradiation of glioma-bearing mice injected with the BCDs-exosomes resulted in tumor mass disappearance and prolonged survival.

A different possible strategy is the use of cell membrane fragments to cover boron-containing nanomaterials whose use is hampered by poor dispersibility and short blood circulation time, as in the case of BNNS [173]. Indeed, some authors proposed a system composed of BNNS, synthesized by a chemical vapor deposition method, covered with red blood cell (RBC) membranes, obtaining the so-called camouflaged BNNS (CM-BNNS) [119]. The RBC ghosts, isolated by sequential centrifugation steps from RBC, were mixed and extruded with the BNNS. The effective RBC membrane covering was verified by TEM observation and by size and zeta potential measurement through DLS: the hydrodynamic diameter decreased from 760 nm of nude BNNS to 146 nm of CM-BNNS, confirming the aggregation phenomena evidenced by TEM, while the zeta potential changed from −32.3 mV to –10.6 mV. Cytotoxicity tests performed in vitro on HEK 293 and HeLa cells confirmed that the RBC membrane coating improved BNNS biocompatibility, as also assessed by the in vivo biodistribution studies performed in mice, resulting in prolonged blood circulation and no significant liver and kidney toxicity.

## 3. Critical and Positive Aspects of the Nanosized Technologies

The described technologies are affected by some limits, as already underlined by Carlsson et al. [174]: the amount of ^10^B atoms per tumor cell necessary for an effective treatment is approx. 10^9^, therefore, only the technologies able to deliver such an amount may aspire to find application in real-world therapy; besides, the pharmacokinetics of each nanosized vehicle should be deeply understood before testing in humans, to rule out those possibly exposing the patient to boron accumulation in normal tissues. Another challenge related to BNCT is the search for techniques for in vivo quantitation of ^10^B and of the released Li and α particles in tissues during neutron irradiation. Finally, in the case of brain tumors, the ability of the nanosystem to cross the BBB should be evaluated in the early development stages and possibly improved with appropriate decorations. From these perspectives, limitations related to some of the described works are the in vivo radiation dosimetry and the limited penetration of the nanosystems into high-mass tumors.

Each nanotechnology overviewed in this paper boasts signature pros: liposomes have been extensively studied, have rather high drug loadings, and may be manufactured on large scale; iron oxide NPs allow easy localization and may benefit from physical targeting; gold NP may be exploited for combined PTT-NBCT therapy; silica NP may be obtained with specific features, such as size and porosity; very small NP such as QD and B_4_C particles easily cross cell membranes; polymeric derivatives let facile chemical modifications or provide chemical handles for targeting moiety attachment; finally, biologically-derived vesicles may provide natural active targeting features, if the manufacturing process does not alter their protein composition.

Generally, when discussing nanoscaled particles, it is necessary to consider some toxicity issues, which depend on their size, shape, chemical composition, and extent of agglomeration. These include possible side accumulation in the liver, spleen, lungs, and kidneys, and the oxidative stress, which might be related to iron oxide and gold NPs exposure, as well as mitochondrial dysfunction and DNA damage. Moreover, data on the systemic distribution and the toxic effects following chronic exposure to elemental boron and boron carbide NPs are currently lacking.

Therapeutics production costs and scalability to industrial processes are other concerns in nanomedicine. In particular, standard and harmonized procedures for the production of some types of nanosystems are still insufficient, causing in some cases (i.e., cell-derived vesicles) a high batch-to-batch variability of the final product.

## 4. Conclusions

Boron neutron capture therapy is a promising cancer treatment, endowed with selectivity and non-invasiveness. Several clinical trials employing the boronated compounds BSH and BPA-fr have been conducted in the past years and are currently ongoing, but there is an urgent need for new boronated agents with improved pharmacokinetic properties and higher tumor selectivity and retention, allowing the accumulation of larger amounts of boron in the target tissues. A possible strategy to accomplish these improved features resides in the development of nanoparticulate drug delivery systems, usually characterized by high drug loading values and allowing for versatile functionalization, to achieve the targeting towards specific cancer hallmarks. Indeed, the use of NPs as boron carriers might ease the overcoming of the issue related to the need for a minimum concentration of boron atoms at the target site, for BNCT to be effective.

Among the nanosystems developed for BNCT agents vehiculation reviewed in this paper, liposomes seem to be the most investigated ones, due to their ease of preparation and surface functionalization, along with their biocompatibility. In general, the development of targeted liposomal systems appears the best option, since they allow active targeting, which may improve tumor/normal tissue distribution, and are endowed by high drug loading capacity, letting to include also anticancer agents.

The use of cell-derived nanosystems is another promising approach, which was explored only by a few researchers; indeed, even if this type of system allows to overcome biocompatibility issues, the isolation of cell membrane-derived vesicles or cell membrane fragments is affected by high batch-to-batch variability and harmonized guidelines for the purification of these materials are still not available. Noteworthy, new technologies aiming at the development of hybrid systems are being investigated, combining the advantages of homing ability and biocompatibility of cell-derived vesicles with the higher yields shown by synthetic NPs manufacturing processes.

The studied technologies acquire value when tested in vivo after irradiation; however, most of the cited works do not provide this kind of data, probably because of the difficulty for researchers to get access to neutron irradiation facilities. This issue might be one of the causes delaying the clinical phase testing of boron-vehiculating nanosystems.

## Figures and Tables

**Table 1:** Liposomal formulations for BNCT applications.

Ref.	Functionalization for Targeting	B Derivative	Physical Features: Size/Drug Loading/Encapsulation Efficiency	Assays	BNCT Efficacy Assays
[46]		Na_2_B_10_H_10_; Na_2_B_12_H_11_SH; Na_2_(n-B_20_H_18_); Na_2_(i-B_20_H_18_); K_4_B_20_H_17_0H; Na_3_B_20_H_19_	43–70 nm/-/2–3%	Ex vivo: EMT6 mammary adenocarcinoma-bearing BALB/c mice	
[47]		K[*nido*-7-CH_3_(CH_2_)_15_-7,8-C_2_B_9_H_ll_]	42–114 nm/-/53–80%	Ex vivo: EMT6 mammary adenocarcinoma-bearing BALB/c mice	
[48]		Na_3_[1-(2′-B_10_H_9_)-2-NH_3_B_10_H_8_] + K[*nido*-7-CH_3_(CH_2_)_15_-7,8-C_2_B_9_H_ll_]	57–114 nm/-/-	Ex vivo: EMT6 mammary adenocarcinoma-bearing BALB/c mice	
[49,50]		Na_3_[1-(2′-B_10_H_9_)-2-NH_3_B_10_H_8_] + K[*nido*-7-CH_3_(CH_2_)_15_-7,8-C_2_B_9_H_11_]	109–134 nm/-/-	Ex vivo: EMT6 mammary adenocarcinoma-bearing BALB/c mice	
[51]		*O*-closocarboranyl *β*-lactoside; 1-methyl-*o*-carboranyl-2-hexylthioporphyrazine	140–450 nm/-/-	In vitro: DHD/K12/TRb rat colon carcinoma and B16-F10 murine melanoma cells	
[52]	Internalizing-RGD	Doxorubicin conj. with 1-bromomethyl-*o*-carborane	150–225 nm/-/-	In vitro: GL261 glioma cancer cells; in vivo: GL261-bearing C57BL/6 mice	In vivo and ex vivo on mice: survival curve and average weight change curve, histopathological analysis
[53]		BPA	-/-/-		
[54]		BPA-fructose; BPA-fructose+BPA	156–195 nm/-/52–82%	Ex vivo: liver metastases-induced BD-IX strain rats	
[55]		BPA-fructose; 2-nitroimidazole derivative B-381	134–140 nm/1317–1801 ppb/4.5–5%	In vivo: bilateral D54 glioma-bearing athymic nude mice	
[56]		BSH	98–109 nm/-/2.5–3.4%	In vivo: male NIH-Swiss mice	
[57]	Anti-human CEA monoclonal antibody	Cs_2_^10^B_12_H_11_SH	-/-/-	In vivo: AsPC-1 human pancreatic carcinoma cells; in vivo: AsPC-1-bearing male BALB/c nu/nu mice	
[58]		Cs_2_^10^B_12_H_11_SH	-/-/ -	In vivo: MRK nu/nu-1 human breast cancer cells	
[59]	Transferrin	BSH	107–123 nm/26–30 µg/µmol lipid/6–8%	In vivo: colon-26 mouse colon carcinoma cells; ex vivo: colon-26-bearing male BALB/c mice	In vivo on mice: survival and tumor growth rate
[60]	Rat or mouse anti-EGFR antibody	BSH	130 nm/-/-	In vivo: U87 ΔEGFR, U87 WT and PAU87 human glioma cells; in vivo: U87 ΔEGFR-bearing BALB/c Slc-nu/nu mice	
[61]		*Closo*-dodecaborate lipids	95–105 nm/-/-	In vivo: colon-26 mouse colorectal carcinoma cells; in vivo: colon-26-bearing female mice	In vivo on mice: tumor volume
[62]		BSH-encapsulating 10% distearoyl boron lipid	127 nm/-/-	In vivo: colon-26-bearing female Balb/c mice	In vivo on mice: tumor volume
[63]		BSH sodium and spermidinium salts, Na_2_[B_12_H_12_], Na_2_[B_12_H_11_OH] and [B_12_H_11_NH_3_] sodium and spermidinium salts	100 nm/2635–13970 ppm/-	In vivo: colon-26 cells mouse colon carcinoma cells; in vivo: colon-26-bearing female mice	
[64]		BSH	104–115 nm/-/2.5–46%		

**Table 2:** Other NPs for BNCT applications, classified by the type of nanocarrier.

Ref.	Functionalization for Targeting	B Derivative	Physical Features: Size/Drug Loading/Encapsulation Efficiency	Assays	BNCT Efficacy Assays
**Elemental Boron NPs**					
[65]		B	5–15 nm/100%/-	In vivo: T98G human glioma cells	In vivo on cells: cell viability
[66]		B	25–28 nm/-/-	In vivo: T98G, U87, and U251 human glioma cells	In vivo on cells: cell proliferation ability
[67]		B	37 nm/-/-	In vivo: HeLa cervical cancer cells	
**Iron Oxide NPs**					
[68]		Boron nitride	10 nm/-/-	In vivo: MCF-7, MCF-10, and HeLa cells	
[69,70]		Carborane borate	9–28 nm/11%/-	In vivo: mouse embryonic fibroblasts	
[71,72]		Isopropyl-*o*-carborane	60 nm/15%/-	In vivo: HeLa, PC-3 (prostate cancer cell), HT-29 (colon cancer cell), BxPC-3 (pancreatic cancer cell), L929 (mouse subcutaneous adipose tissue cells)	
[73]		*M*-carboranylphosphinate and its acid form	8–133 nm/-/-	In vivo: A172 glioblastoma and hCMEC/D3 endothelial cells; in vivo: mice	In vivo on cells: proliferation rate
[74]		B	64 nm/9%/-	In vivo: L929 mouse fibroblasts, 4T1 mammary carcinoma and B16 melanoma cells, human peripheral blood mononuclear cells; ex vivo, in vivo: Balb/c male mice	
[75]		B	15 nm/77%/-	In vivo: U87 human glioblastoma and SW-620 human colorectal adenocarcinoma cells	
[76]		GdBO_3_	20–150 nm/-/-		
[77]	Folic acid	GdBO_3_	20–150 nm/2.5%/-	In vivo: MIA-Pa-Ca-2 human pancreatic cancer, HeLa human cervical carcinoma and A549 human lung carcinoma cells	
[78]		3-(Isopropyl-*o*-carboranyl) hydrindone	110 nm/0.077 mg/g/-	In vivo: PC-3 prostate cancer epithelial cells, BxPC3 pancreatic cancer cells, MCF7 breast cancer cells, HepG2 and L929 murine fibroblast cells, and human skin fibroblasts	
[79]		Di(*o*-carborano-1,2-dimethyl)borate	386 nm/15.4%/-	In vivo: HepG2 cancer cells and human skin fibroblasts	
[80]		B	180–280 nm/3.1 × 10^17^ atoms/mg/-	In vivo: T98G glioblastoma cells	
**Gold NPs**					
[81]		1,2-Dicarba-*closo*-dodecaborane(12)-9-thiol (9SHOCB) and 1,2-dicarba-*closo*-dodecaborane(12)-9,12-dithiol (9,12SH-OCB)	35–74 nm/-/-	In vivo: UMR-106 rat osteogenic sarcoma cells	
[82]		Carborane derivative (NH_2_NH_3_)^+^[7-NH_2_(CH_2_)_3_S-7,8-C_2_B_9_H_11_]^−^	9 nm/-/-	In vivo: HeLa, U87cells and L02 cells; in vivo: HeLa-xenografted nude mice	
[83]	^123^I-labeled anti-HER2	Boron cage-SH	58 nm/-/28–36%	In vivo: N87 human gastric cancer cells; in vivo: male NOD/SCID mouse	
[84]		Thiol B cage, BPA, or BPA fructose	158–395 nm/-/3–35%	In vivo: N87 human gastric cancer male immunodeficient mouse	
[85]	Trastuzumab (pretargeting agent)	Cobalt bis(dicarbollide) anion [3,3′-Co(C_2_B_9_H_11_)_2_]^−^	40 nm/195 µg/mg/-	In vivo: BT-474 breast cancer cells; in vivo: BT-474 breast cancer-bearing immunocompromised NOD/SCID mice	
[86]		Undecahydro-*closo*-dodecaborate (B_12_H_12_)	29 nm/0.307 µg B/mg of gold/-	In vivo: MCF-7 human mammary adenocarcinoma cells; in vivo: U87 glioma-bearing male SCID mice	
**Silica NPs**					
[87]		B	64 nm/42%/-		
[88]	RGD-K	B	90 nm/-/-	In vivo: ALTS1C1 cells; in vivo: ALTS1C1-bearing mice	In vivo on cells: cell viability; ex vivo and in vivo on mice: tumor growth and mouse survival
[89]	Amino-galactoside ligands	*O*-carborane	-/445–539 µg/mg/-	In vivo: HepG2 liver cancer cells	In vivo on cells: colony formation
[90]	Activatable cell penetrating peptide	BSH	200 nm/1.27%/-	In vivo: ALDH+ cancer stem cells sorted from CH-2879 chondrosarcoma cells; in vivo: BALB/c nu/nu male mice	In vivo on cells: DNA damage and clonogenic survival
[91]	cRGD targeting pancreatic tumor sites overexpressing integrin receptors	*O*-carborane	136 nm/141.5 mg/g/-	In vivo: PANC-1 (human) and Panc-2 (mouse) cells; in vivo: female C57BL/6 mice	
[92]		BPA	170 nm/2.5%/-	In vivo: OVCAR8 ovarian cancer cells, A549 lung cancer cells, FaDu head and neck cancer cells; *animal model*: CAM (chorioallantoic membrane) model transfected with human OVCAR8 ovarian cancer cells	
[93]	pH-(low)-insertion peptide	Boric acid	-/190 μg/g/-	In vivo: B16−F10 mouse melanoma and WEHI 164 mouse fibrosarcoma cells; ex vivo, in vivo: WEHI 164 tumor-bearing mice	In vivo on cells: cell viability; ex vivo and in vivo on mice: tumor growth
**Boron Carbide NPs and Quantum Dots**					
[94,95]	TAT peptide		284–459 nm/-/-	In vivo: B16 F10 mouse melanoma cells, B16-OVA cells; ex vivo, in vivo: B16-OVA-bearing C57BL/6J mice	
[96]	Transferrin		279 nm/-/-	In vivo: HeLa human cervical carcinoma and normal rat kidney epithelial cells; ex vivo: tumor-bearing mice	
[97]	Human immunoglobulin G		80 nm/-/-	In vivo: MC38 murine colon carcinoma and RAW 264.7 murine macrophages	
[98]			7 nm/-/-	In vivo: embryonic kidney HEK-293 cells, HeLa cervical cancer cells and human breast adenocarcinoma MCF-7 cells	
[99]	Pretargeting with *trans*-cyclooctene modified trastuzumab		7 nm/3.8%/-	In vivo: human BT-474 breast cancer cells; ex vivo, in vivo: BT-474 xenografted female NOD.CB17-Prkdcscid/J mice	
**Polymeric NPs and Micelles**					
[100]	ACUPA (PSMA-targeting)	*O*-carborane	150–165 nm/1.86%/-	In vivo: PC3-flu and PC3-pip prostatic adenocarcinoma cells; ex vivo, in vivo: PC3-flu and PC3-pip xenografted male athymic mice	
[101,102]		1,2-bis(4-vinylbenzyl)-*closo*-carborane	85 nm/0.26%/3.1%	In vivo: colon-26 cells; ex vivo, in vivo: colon-26 tumor-bearing BALB/c mice	
[103]		Decaborane	108 nm/9.9%/60.0%	Ex vivo: female Sprague−Dawley rats and U14 tumor-bearing female KM mice	Ex vivo and in vivo on U14 tumor-bearing female KM mice: tumor growth and mouse body weight
[104]	Tumor penetrating RGD peptide	BSH	25 nm/3.81%/-	In vivo: A549 human lung cancer cells, B16−F10 mouse melanoma cells, C6 rat glioma cells, 4T1 mouse breast cancer cells and HeLa cervical cancer cells; ex vivo: A549 tumor-bearing BALB/c nude mice and B16F10 tumor-bearing mice	
[105]		BSH	36 nm/-/-	In vivo: C26 mouse colorectal carcinoma and human aortic endothelial cells; ex vivo: C26 tumor-bearing BALB/c mice	Ex vivo and in vivo on C26 tumor-bearing BALB/c mice: tumor growth and mouse body weight
[106,107]		*O*-carborane	100–150 nm/4.8–5.6%/-	In vivo: B16 melanoma cells; ex vivo: B16 melanoma-bearing mice	
[108]		BPA	145 nm/-/> 95%	In vivo: U87MG human glioblastoma cells	In vivo on CR-39 detectors
[109]		Tetraboronated porphyrin	100 nm/-/-	In vivo: mouse melanoma B16-F10 and 4T1 mouse breast cancer cells; ex vivo, in vivo: B16-F10 tumor-bearing C57BL/6 mice and 4T1 tumor-bearing BALB/c mice	Ex vivo and in vivo on B16−F10 tumor-bearing mice
[110]		Carborane	110 nm/11.38%/-	In vivo: 4T1 mouse breast cancer cells; ex vivo, in vivo: 4T1 tumor-bearing mice	In vivo on 4T1 tumor-bearing mice
[111]	Galactose	Carborane	135 nm/3%/-	In vivo: CAL27 oral adenosquamous carcinoma, U251 glioma and HepG2 hepatocellular carcinoma cells	In vivo on HepG2 cells: apoptosis rate
[112]		Carborane-containing arene ruthenium complex	19 nm/-/-	In vivo: A2780 and A2780 cisplatin-resistant human ovarian cancer cells	In vivo on A2780 and A2780cisR cells: cell viability
[113]		Dodecaborate	13–15 nm/-/-	In vivo: U87 human glioblastoma, HeLa cervical cancer and Jurkat cells	
**Other NPs**					
[114]	Folic acid	Boron oxide	20–50 nm/8% mol B/NP/-	In vivo: HeLa cervical cancer cells	In vivo on HeLa cells: cell viability
[115]	Folic acid		255 nm/9.8%/-	In vivo: erythrocytes and platelets from human plasma specimens, MO7e human megakaryoblastic leukemia cells, DHD colon carcinoma cells, UMR osteosarcoma cells	
[116]			5 nm/30% of boron atoms/NP/-	In vivo: on ALTS1C1 mouse astrocytes	In vivo on cells: cell viability
[117]		Phenyl-^10^B-boronic acid	53–67 nm/2%/-	In vivo: CT26 murine colon tumor cells; ex vivo, in vivo: CT26 tumor-bearing BALB/c mice	In vivo on mice: tumor growth and body weight
**Exosomes and Biomimetic Vesicles**					
[118]		Boron-containing carbon dots	97 nm/17.90%/85.24%	In vivo: MDA-MB-231 human breast cancer, U251 glioma and HepG2 hepatocellular carcinoma cells; ex vivo, in vivo: BALB/c nude mice, U-87 glioma-bearing mice	In vivo on mice: tumor growth and body weight
[119]			146 nm/-/-	In vivo: HEK 293 human embryonic kidney and HeLa cervical cancer cells; ex vivo: female Kunming mice	

## Abbreviations

1-MNPs: boron cluster-magnetic nanoparticle nanohybrids; ACPP: cell penetrating peptide; ACUPA: 2-(3-((S)-5-amino-1-carboxypentyl)ureido) pentanedioic acid; APTES: (3-aminopropyl)triethoxysilane; APTMS: (3-aminopropyl)trimethoxysilane; AuNCs: gold nanoclusters; AuNPs gold nanoparticles; BA: boric acid; BCDs: boron-containing carbon dots; BDDs: boron-doped carbon dots; BC-EG-SH: boron cage functionalized-ethylene glycol-SH chain; BCNO NPs: boron carbon oxynitride nanoparticles; B-HSA: undecahydro-*closo*-dodecaborate-HSA conjugate; BNCT: boron neutron capture therapy; BNNS: boron nitride nanospheres; BNPs: boron cluster-containing redox nanoparticles; BPA: boronophenylalanine; BPA-fr: BPA complex with fructose; BPMO NPs: biodegradable periodic mesoporous organosilica nanoparticles; BPN: boronated porphyrin-loaded poly(lactide-co-glycolide)-monomethoxy-poly(polyethylene-glycol) nanocomplex; BSH: mercaptoundecahydro-*closo*-dodecaborate; BX: borax; CA: citric acid; CAM: chorioallantoic membrane; CB: carborane; CL micelles: cross-linked micelles; CM-BNNS: camouflaged boron nitride nanospheres; COSAN: cobalt bis[dicarbollide]; cRGD-PEI: cRGD-modified polyethyleneimine polymer; CS: chitosan; DFB: deferoxamine B; DLS: dynamic light scattering; DMSNs: dendritic mesoporous silica nanospheres; DNDs: detonation nanodiamonds; DOGS-NTA-Ni: 1,2-dioleoyl-*sn*-glycero-3-[(N-(5-amino-1-carboxypentyl)iminodiacetic acid)-succinyl] nickel; DOPA: 1,2-dioleoyl-*sn*-glycero-3-phosphate; DOPC: 1,2-dioleoyl-*sn*-glycero-3-phosphocholine; DOPE: 1,2-dioleoyl-*sn*-glycero-3-phosphoethanolamine; DOPG: 1,2-dioleoyl-*sn*-glycero-3-phospho-(1′-rac-glycerol); DOTAP: 1,2-dioleoyl-3-trimethylammonium-propane; DOX-CB: complex of doxorubicin with 1-bromomethyl-*o*-carborane; DPPC: 1,2-dipalmitoyl-sn-glycero-3-phosphocholine; DSBL lipid: BSH-conjugated DSPC; DSPC: 1,2-distearoyl-sn-glycero-3-phosphocholi; DSPE-PEG: 1,2-distearoyl-sn-glycero-3-phosphoethanolamine-N-[amino(polyethyleneglycol)-2000]; DTPA: diethylenetriamine penta-acetic acid; EDC: 1-ethyl-3-(3-dimethylaminopropyl)-carbodiimide; EE: encapsulation efficiency; EGFR: epidermal growth factor receptor; EVs: extracellular vesicles; FACS: fluorescence activated cell sorting; FITC-SiO_2_: fluorescein isothiocyanate-doped silica; FTIR: Fourier transform infrared; GM1: monosialoganglioside; H2Pz-COB: 1-methyl-*o*-carboranyl-2-hexylthioporphyrazine; HEC: hydroxyethyl cellulose; HER2: anti-human epidermal growth factor receptor-2; HSA: human serum albumin; ICP-MS: inductively coupled plasma mass spectrometry; ICP-OES: inductively coupled plasma optical emission spectrometry; iRGD: internalizing-RGD peptide; LCOB: *o*-closocarboranyl β-lactoside; mAb: monoclonal antibody; MIP-MS: microwave-induced plasma mass spectrometer; mPEG-b-P(LA-co-MPC): mPEG-b-poly(lactic acid-co-5-methyl-5-propargyloxycarbonyl-1,3-dioxan-2-one; MRI: magnetic resonance imaging; MSN: mesoporous silica nanoparticles; NBD-cholesterol: 25-[*N*-[(7-nitrobenz-2-oxa-1,3-diazol-4-yl)-methyl]amino]-27-norcholesterol; NCL micelles: non-cross-linked micelles; NPs: nanoparticles; PCCL: polycarboxycaprolactone; PCMS: poly(chloromethylstyrene); pDNA: CD47 gene-targeted CRISPR-Cas9 gene knock-out plasmid; PEG-b-PLA: PEG-block-poly(D,L-lactide-co-glicolide); PEG-b-PLGA: PEG-block-poly(D,L-lactide-co-glycolide); PEI: polyethyleneimine; PEO-b-PCL: poly(ethyleneoxide)-b-poly(caprolactone) diblock-copolymer; PEO-b-PGEA: poly(ethyleneoxide)-block-poly(2-(N,N,N′,N′-tetramethylguanidium)ethyl acrylate); PET: positron emission tomography; PET-CT: positron emission tomography-computed tomography; PLA: poly(D,L-lactide); PLGA: poly(D,L-lactide-co-glicolide); PLGA−mPEG: poly(lactide-co-glycolide)-monomethoxy-poly(ethylene-glycol); PLLGA: poly(L-lactide-co-glycolide); PLNPs: persistent luminescence nanoparticles; PSMA: prostate specific membrane antigen; PTT: photothermal therapy; PVP: polyvinyl pyrrolidone; RBC: red blood cells; SEM: scanning electron microscopy; SiNPs: silica nanoparticles; S-NHS: *N*-hydroxysulfosuccinimide; SPIONs: superparamagnetic iron oxide nanoparticles; TAPP: 5,10,15,20-tetrakis(4-aminophenyl)porphyrin; TAT: transactivator of transcription; TCO: trans-cyclooctene; TEM: transmission electron microscopy; TF: transferrin; TSL: temperature-sensitive liposomes; Tz: tetrazine; UNDCA: 10-undecenoic acid; XRD: X-ray diffraction.
